# *Tviblindi* algorithm identifies branching developmental trajectories of human B‐cell development and describes abnormalities in RAG‐1 and WAS patients

**DOI:** 10.1002/eji.202451004

**Published:** 2024-09-05

**Authors:** Marina Bakardjieva, Ondřej Pelák, Marjolein Wentink, Hana Glier, David Novák, Jitka Stančíková, Daniela Kužílková, Ester Mejstříková, Iga Janowska, Marta Rizzi, Mirjam van der Burg, Jan Stuchlý, Tomáš Kalina

**Affiliations:** ^1^ CLIP Department of Paediatric Haematology and Oncology Second Faculty of Medicine Charles University Prague Czech Republic; ^2^ Department of Paediatric Haematology and Oncology University Hospital Motol Prague Czech Republic; ^3^ Department of Internal Medicine Erasmus MC University Medical Center Rotterdam Rotterdam the Netherlands; ^4^ Department of Applied Mathematics Computer Science and Statistics Ghent University Ghent Belgium; ^5^ Data Mining and Modeling for Biomedicine Center for Inflammation Research VIB‐UGent Ghent Belgium; ^6^ Department of Rheumatology and Clinical Immunology Freiburg University Medical Center University of Freiburg Freiburg Germany; ^7^ Center for Chronic Immunodeficiency University Medical Center Freiburg Faculty of Medicine University of Freiburg Freiburg Germany; ^8^ Department of Pediatrics Laboratory for Pediatric Immmunology Leiden University Medical Center Leiden the Netherlands

**Keywords:** B‐cell development, Mass cytometry, Trajectory inference, CD73, RAG‐1, WAS

## Abstract

Detailed knowledge of human B‐cell development is crucial for the proper interpretation of inborn errors of immunity and malignant diseases. It is of interest to understand the kinetics of protein expression changes during development, but also to properly interpret the major and possibly alternative developmental trajectories. We have investigated human samples from healthy individuals with the aim of describing all B‐cell developmental trajectories. We validated a 30‐parameter mass cytometry panel and demonstrated the utility of “*vaevictis*” visualization of B‐cell developmental stages. We used the trajectory inference tool “*tviblindi*” to exhaustively describe all trajectories leading to all developmental ends discovered in the data. Focusing on Natural Effector B cells, we demonstrated the dynamics of expression of nuclear factors (PAX‐5, TdT, Ki‐67, Bcl‐2), cytokine and chemokine receptors (CD127, CXCR4, CXCR5) in relation to the canonical B‐cell developmental stage markers. We observed branching of the memory development, where follicular memory formation was marked by CD73 expression. Lastly, we performed an analysis of two example cases of abnormal B‐cell development caused by mutations in RAG‐1 and Wiskott–Aldrich syndrome gene in patients with primary immunodeficiency. In conclusion, we developed, validated, and presented a comprehensive set of tools for the investigation of B‐cell development in the bone marrow compartment.

## Graphical abstract

Novel computational framework *tviblindi* identifies branching B cell developmental trajectories in healthy (HD) and abnormal (RAG‐1 and Wiskott–Aldrich syndrome primary immunodeficiency) bone marrow and peripheral blood samples using 30 parameter mass cytometry. Integrated *vaevictis* projection allows interpretation of trajectories to *κ* (grey arrow) and *λ* (black arrow) light chain expressing B cells, memory development branching trajectories (solid vs. dashed arrows), and abnormal trajectories caused by RAG‐1 and WAS gene mutations. The pseudotime line plot shows the dynamics of selected marker expressions along the trajectories. Created with BioRender.com.

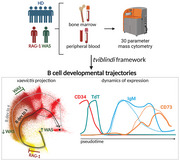

## Introduction

B‐cells, together with T‐cells, are adaptive immunity constituents responsible for antigen‐specific responses and immune system memory. Mature and terminally differentiated B‐cells produce high‐affinity antibodies. B‐cells develop from hematopoietic stem cells in the bone marrow, exit to peripheral blood, enter the secondary lymphoid organs upon antigen encounter to mature in the germinal center and recirculate to peripheral blood and eventually home back to the bone marrow as antibody‐secreting cells.

These principles, key developmental stages, and molecular mechanisms are largely known and surface molecule expression defining the immunophenotype of each stage is published extensively [1].

B‐cell‐development abnormalities or complete blocks are found as a result of monogenic lesions in primary immunodeficiency disorders (PIDD) [2]. Leukemia and lymphoma of B‐cell origin is the most common neoplasia in children, defects in B‐cell development and regulation are frequent causes of immune dysregulation diseases in children and adults, making the B‐cell development and function an attractive therapeutic target. As B‐cell targeted therapies (e.g. anti‐CD20 monoclonal antibodies, anti‐CD19 CAR‐T) become available and their usage increase, iatrogenic B‐cell developmental failures are becoming common conditions [3].

However, our understanding of the particularities of B‐cell developmental abnormalities in those conditions is still incomplete. We currently lack detailed knowledge of the dynamics of additional, noncanonical molecules (new phenotype markers, signaling molecules, therapeutic targets, *in vivo* response to therapy markers). Second, we lack detailed insight into within‐a‐stage changes, details of transitions, or intermediate stages. Third, we lack tools to disclose additional, alternative, or nondominant trajectories potentially present in human patients.

Recent advances in single‐cell analysis extended the capabilities of clinical flow cytometry beyond 10 parameters, and in another quantum leap forward, spectral [4] or mass cytometry [5] enabled us to investigate 40 parameters on each cell [6]. In a proof of principle work of Bendall et al. [7], the Wanderlust algorithm was applied to B‐cell developmental mass cytometry data, showing the assembled progression of markers in a single pathway. We have previously proven that a single 10‐color flow cytometry tube is capable of describing the crucial stages of B‐cell development and its abnormalities found in PIDD with monogenic lesions in the scope of the EuroFlow consortium standardized protocol [8]. This knowledge is essential since the inherent assumption of a single‐cell trajectory inference is that data contain all markers needed to distinguish all stages and their transition points. Recently, Saelens et al. [9], benchmarked 45 trajectory inference algorithms out of 70 available, concluding that only several would allow for multiple endpoints discovery. Most are built for single‐cell RNA data, where the number of cells analyzed is low (10,000) but the number of parameters is high, which contrasts with the mass cytometry dataset, where tens of millions of cells are analyzed with several dozens of parameters. Our objective was to limit the amount of prior information to the starting cell subset, generate all putative random walks, and allow for their graphical and user‐friendly interrogation and in‐depth analysis of the selected trajectories.

In the current study, we set out to develop a mass cytometry protocol and analytical tools that would allow us to interrogate the B‐cell developmental pathways in the bone marrow in more detail. We use the 10‐color EuroFlow tube as a benchmark. We interrogate the pathways of development leading to terminal cell types expressing either κ light chain or *λ* light chain across two tissue types.

## Materials and methods

### Sample cohort composition and preparation

Fresh human bone marrow samples (*n* = 4) were obtained from pediatric patients with excluded hematological disease or immunological disorder or (*n* = 1) from fully recovered patients 1 year after successful B‐cell precursor leukemia therapy. Only leftover part of the clinical material was used where Informed consent was given. The study was conducted within a project approved by Motol University Hospital's ethical board. B cells were isolated from the samples using RosetteSep Human B‐cell Enrichment Cocktail (Stemcell Technologies) following the manufacturer's instructions. Isolated bone marrow B cells and precursors were cryopreserved in fetal bovine serum containing 10% DMSO in liquid nitrogen. Cryopreserved bone marrow samples without pre‐enrichment were used from patients with PIDD (RAG1 compound heterozygot c.256_257delAA (p.Lys86ValfsX33), c.2210G>A (p.Arg737His), female, age 1 years, and Wiskott–Aldrich syndrome (WAS) hemizygot c.397G>A, male, age 1.5 months). Peripheral B cells were isolated using the same method either from fresh human peripheral blood (*n* = 2) or from buffy coats (*n* = 3), washed with MaxPar Cell Staining Buffer (Standard BioTools), and used immediately for staining. Bone marrow B cells were thawed for 1 min in a 37°C water bath, rested for 30 min in RPMI medium at 37°C in an incubator, and washed. Individual samples were barcoded [10] with a combination of anti‐CD45 and anti‐HLA‐I metal‐tagged antibodies listed in Supporting Information Table S1, as described previously [11], pooled and further processed in individual tubes.

### Sample staining and acquisition

Metal‐tagged antibodies were either purchased (Standard BioTools) or conjugated in‐house using Maxpar X8 Antibody Labeling Kit (Standard BioTools) according to the manufacturer's instructions. Antibodies were validated and titrated for the appropriate concentrations and are listed in Supporting Information Table S1. The samples were stained as described previously [12] and according to the MaxPar Nuclear Antigen Staining with Fresh Fix (Standard BioTools) protocol as described by the manufacturer. Mass cytometry sample acquisition was performed on Helios and CyTOF XT instruments (Standard BioTools, CyTOF 6.7.1014 and 8.0 software) after preparation according to the manufacturer's recommendation. Flow cytometry measurement of B‐cell precursors was performed exactly as in Wentink et al. [8].

### Data analysis

Acquired samples were exported into FSC format and analyzed manually using sequential bivariate gating in FlowJo (v10.5, FlowJo LLC) software. First, we gated nucleated cells positive for DNA intercalator tagged with 191/193Ir and next the cells positive for particular CD45 and MHC‐class I antibody reagent combinations were gated to resolve the barcodes of the individual bone marrow or peripheral blood samples. Next, cell populations for both mass and flow cytometry panels were defined as described previously [8], [13], [14], gating strategy is shown in Supporting Information Fig. S1 and S2. When markers differently expressed by subsets were sought, we used “population comparison” tool in FlowJo, with probability binning and Cox chi‐square statistics. The raw FCS dataset is available on GitHub https://github.com/tomas‐kalina/B‐cell‐developmentFCS.

### Projection with *vaevictis*

For visualization of the mass cytometry data, we used a deep learning‐based dimensionality reduction technique using the *vaevictis* model [15], one of the autonomous modules integrated into our recently developed computational framework *tviblindi*. For the projection, healthy bone marrow (*n* = 4) and peripheral blood (*n* = 4) samples were manually debarcoded and exported as individual FCS files. Next, only cells defined as CD34^+^ or CD19^+^ were concatenated into one FCS file and subsequently used for training the *vaevictis* algorithm, where all panel markers were used for the calculation. Such a trained *vaevictis* model was then applied separately to either the set of bone marrow or the set of peripheral blood cells.

### Trajectory inference in *tviblindi*

For trajectory inference (TI), we used our recent framework called *tviblindi* [15], an algorithm integrating several autonomous modules — pseudotime inference, random walk simulations, real‐time topological classification using persistence homology, and autoencoder‐based 2D visualization using the *vaevictis* model. For the TI, the same concatenated FCS file as for the *vaevictis* projection was used. As a point of origin, stem cells (CD19^‐^CD79a^‐^TdT^‐^CD34^+^) were used. In total, 5000 random walks were probed across the single cell space. Endpoints for further investigation were selected in *tviblindi* graphical user interface (GUI). Topological landmarks were selected in the persistence homology graph in the GUI. Next, walks clustering together were selected on the dendrogram of persistence homology and visually inspected on the *vaevictis* plot. The pseudotime vs. marker line plots were created and exported from the *tviblindi* GUI. For manual analysis of the data in FlowJo, an enhanced FCS file was exported from the *tviblindi* GUI containing all calculated parameters.

## Results

In order to study bone marrow human B‐cell development, we designed a 30‐parameter mass cytometry panel allowing for simultaneous measurement of B‐cell‐specific phenotypic surface markers and functional intracellular proteins (Supporting Information Table S1). We validated the correct assignment of the B‐cell precursor subsets by the Euroflow 10‐parameter flow cytometry diagnostic panel [8]. We found a similar distribution of B‐cell subsets (gated as in Supporting Information Fig. S1) measured in four bone marrow samples by mass cytometry and Euroflow flow cytometry, confirming that the mass cytometry panel can describe the basic stages of the B‐cell development in the bone marrow (Supporting Information Fig. S3).

**Figure 1:**
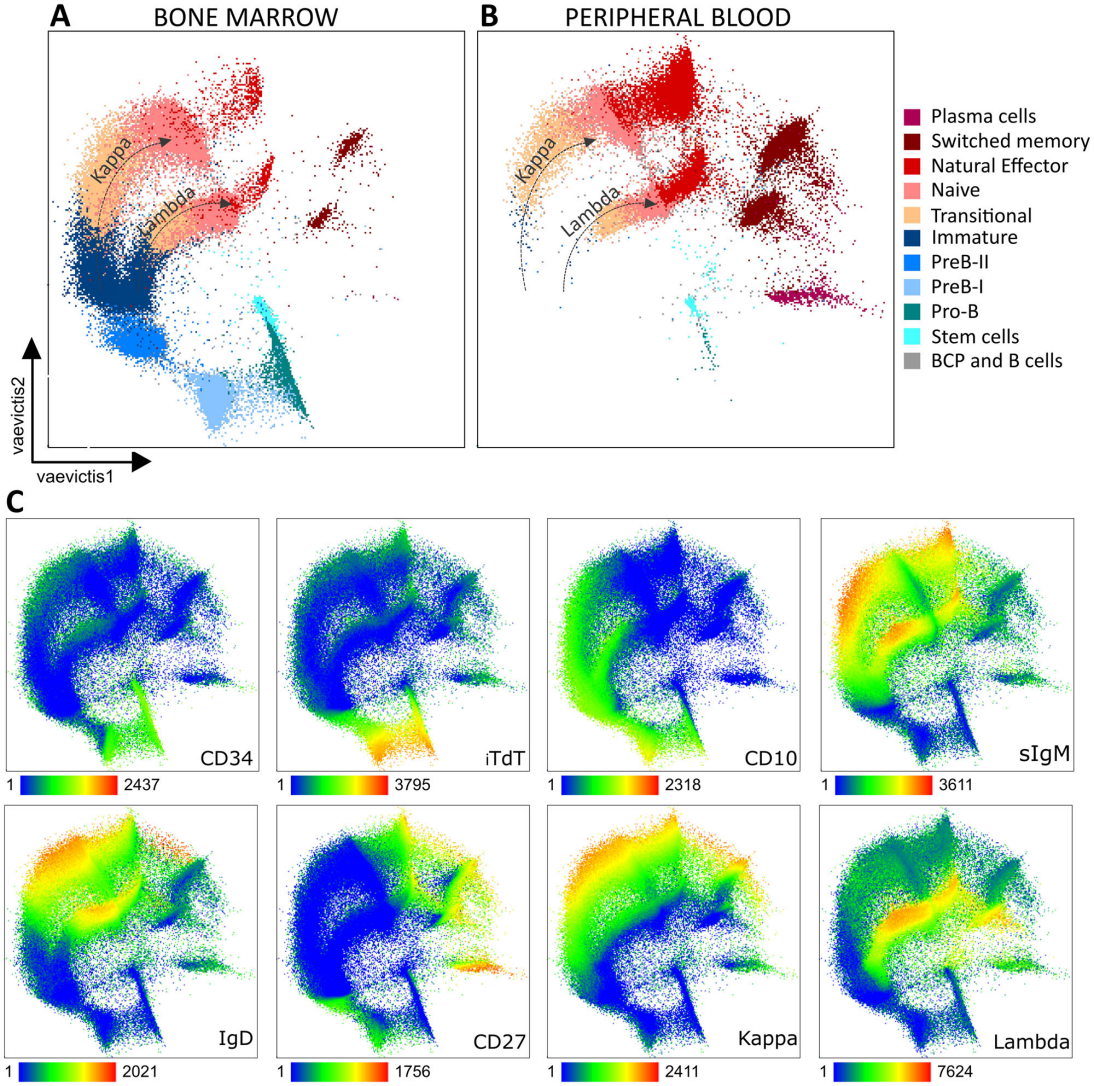
*Vaevictis* dimensionality reduction of B‐cell precursors (BCPs) and B cells in bone marrow and peripheral blood. (A) Healthy bone marrow (*n* = 4, concatenated) and (B) peripheral blood (*n* = 4, concatenated) BCPs and B cells with manually gated populations were applied to the visualization in color, with annotation and counts of the individual subsets. Dotted arrows highlight kappa and lambda B cells (compare to panel C) (C) Visualization of the entire merged data with the expression of chosen canonical markers using a heatmap color gradient where green represents the lowest expression and red the highest.

Next, we visualized the B‐cell precursor subsets using *vaevictis*, a representation learning dimensionality reduction tool built for development visualization. We could observe that the expected main features of the B‐cell precursor to mature B‐cell development were apparent in four bone marrow and four peripheral blood samples (Fig. 1). *Vaevictis* plots of all of the samples individually can be found in Supporting Information Fig. S4. Using information from all 30 markers, *vaevictis* positioned the mature B‐cells adjacent to the B‐cell precursors (Fig. 1A and B), where the aforementioned gated subsets were ordered from the progenitors to mature cell types. Also, the light chain expression highlighted the κ and λ branching (Fig. 1C). Progression of the canonical markers (CD34, TdT, CD10, surface IgM (sIgM), κ chain, λ chain, IgD, and CD27) in the plot corresponds with the expected course of B‐cell development (Fig. 1C).

Thus, the B‐cells and their precursors measured by mass cytometry panel and visualized using *vaevictis* provided bases for interpretation of the putative trajectories of B‐cell development.

**Figure 2:**
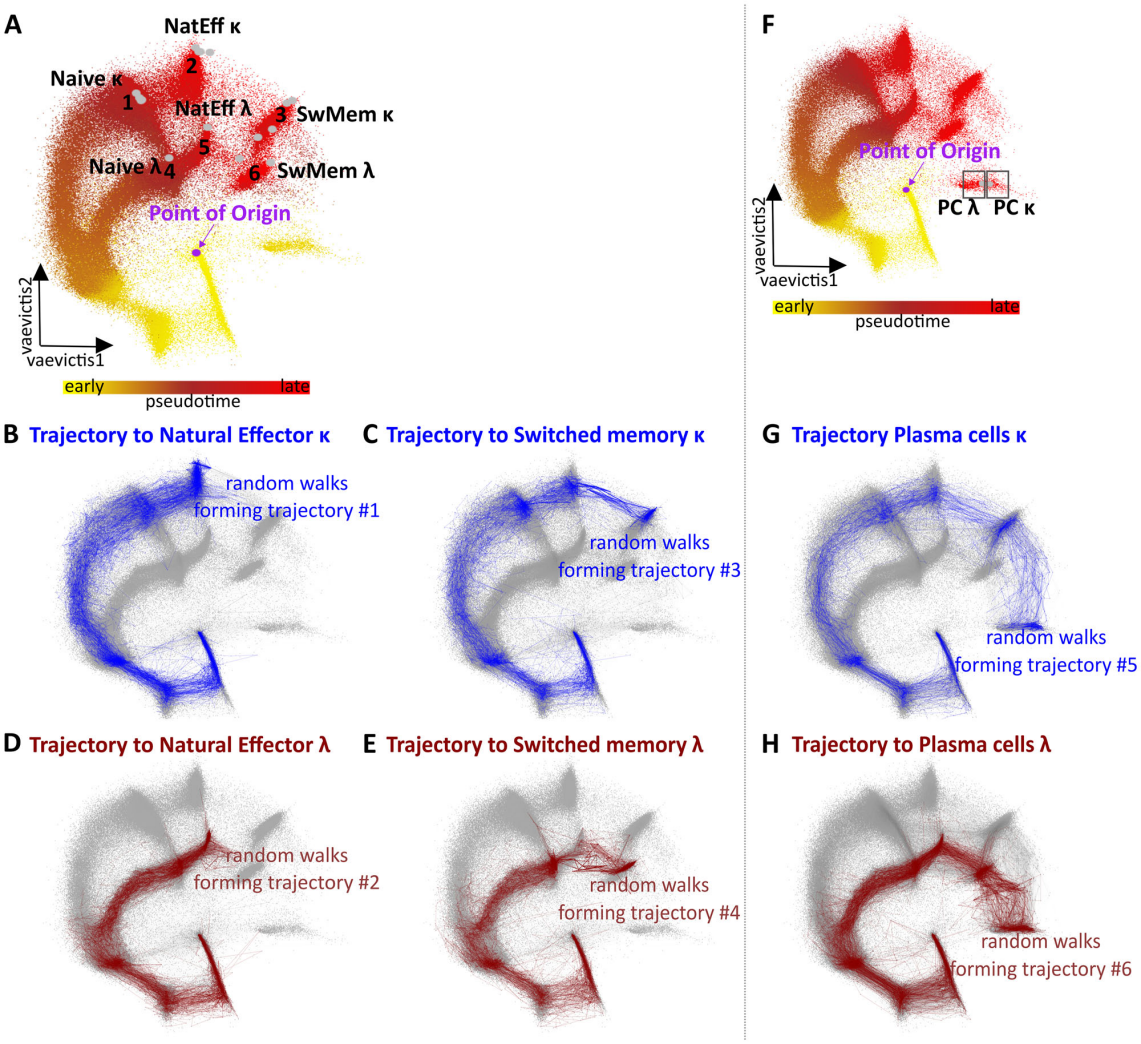
B‐cell developmental endpoints and trajectories leading to Natural Effector and Switched memory κ and λ B cells constructed by *tviblindi*. (A) Groups of endpoints (1–6) represented as gray dots are located at naïve (1;4), natural effector (2;5), and switched memory (3;6) cells in the *vaevictis* plot colored by pseudotime. Yellow color indicates the earliest pseudotime and bright red color indicates the latest pseudotime. The purple dot indicates the point of origin at gated CD34^+^ Stem cells. *Vaevictis* plot with displayed trajectories to (B) natural effector *κ* and (D) *λ* cells and (C) switched memory *κ* and (E) *λ* cells constructed by *tviblindi* (the selection of random walk groups shown in Supporting Information Fig. S5 and S6). (F) Expert‐added endpoints located at Plasma cells (PC) are shown as square gates PC *κ* and PC *λ*, with pseudotime recalculation (in color) on the *vaevictis* plot. Trajectories to (G) PC *κ* and (H) PC *λ* are displayed on the *vaevictis* plot (the selection of random walk groups shown in Supporting Information Fig. S7).

On the concatenated dataset we selected the developmental point of origin at CD34^+^ stem cells. The *tviblindi* algorithm [15] was tasked to construct 5000 random walks directed away from the origin (CD34^+^ stem cell) with respect to the calculated pseudotime on the nearest neighbor graph (KNNg) of all single‐cell events. As the KNNg is directed by the pseudotime, endpoints are automatically detected when a random walk reaches a vertex (single‐cell event) with no out‐going edges. Sixteen endpoints were located in the 6 B‐cell subsets corresponding to mature naive (alternatively PreGC), natural Effectors (alternatively unswitched memory or MZ‐like), and switched memory B cells expressing either *κ* or *λ* light chain (Fig. 2A). We have selected all endpoints leading to each subset individually. Next, we assembled random walks into different coherent trajectories leading to natural effector *κ* and *λ* (Fig. 2B and C) and to switched memory *κ* and *λ* (Fig. 2D and E) on dendrograms of groups of walks, grouped with respect to the persistent homology classes (Supporting Information Fig. S5 and S6). In parallel, we visualized them on the *vaevictis* plot. Since *tviblindi* algorithm and *vaevictis* visualization operate independently in the dataset, we have prioritized abundant walks with particular topology in all dimensions (selected on persistent homology diagram and dendrogram) and those that were transiting through expected cell subsets in a logical sequence (compare Fig. 2B–E with Fig. 1A). Upon expert analysis, we noticed that Plasma cells were not detected as an endpoint. This was caused by the inability of the antibody panel to adequately capture the long distance between Stem cell and Plasma cells, finding an illegitimate pseudotime shortcut instead (note the color gradient in Fig. 2A). We modified the pseudotime calculation in *tviblindi* and introduced the option to set the endpoints manually. This modification allows investigation of the relationship between any two sets of cells within the data. We, therefore, expertly chose and set the Plasma cells *κ* and *λ* as additional endpoints (square gates, Fig. 2F) and thus forced the pseudotime inference to follow the full development accordingly (yellow to red gradient, Fig. 2F) and to detect additional trajectories from Stem cells to Plasma cells (Fig. 2G and H, Supporting Information Fig. S7).

**Figure 3:**
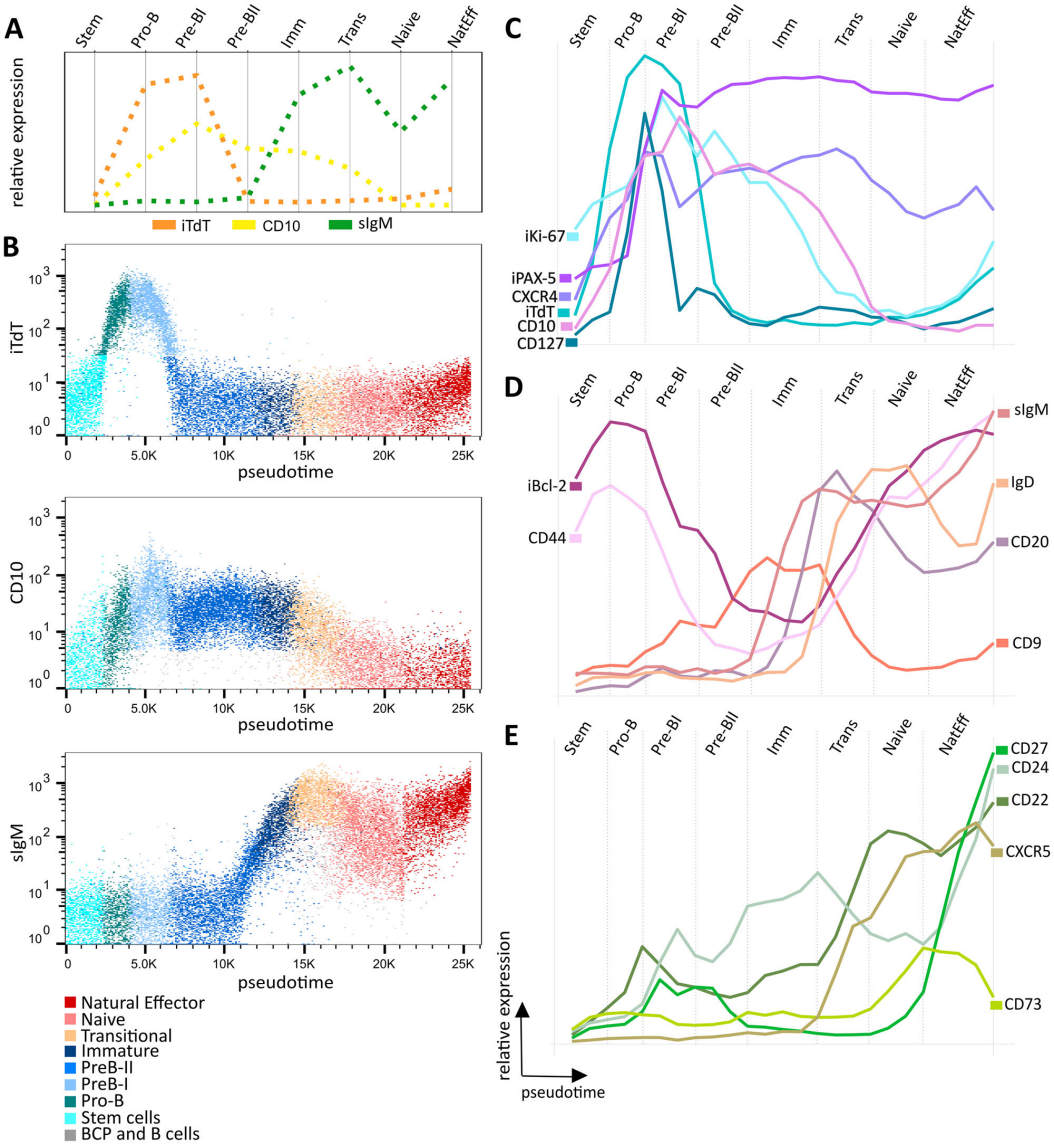
Detailed analysis of the trajectory leading to natural effector κ cells. (A) Median expression of iTdT (orange), CD10 (yellow), and sIgM (green) from manually gated populations. (B) Expression of iTdT (top), CD10 (middle), and sIgM (bottom) on the pseudotime vs. marker dot plots with manually gated populations overlaid in color. Pseudotime line plots showing the average expression of markers upregulated in the (C) early, (D) mid, and (E) late phases of the development, manually annotated by the gated stages.

For further analysis, we selected the trajectory leading to natural effector *κ*. We aimed to investigate changes in expression of the markers along the selected developmental trajectory manually. The *tviblindi* interface (GUI, Supporting Information Fig. S8) allowed us to add all calculated parameters (*vaevictis* 1, *vaevictis* 2, pseudotime, cell assignment to trajectory) and manually investigate the enhanced FCS file for the expression of selected markers along the pseudotime of the selected trajectory (κ, λ and iTdT markers shown; Supporting Information Fig. S9). Next, we aligned the relative expression values of iTdT, CD10, and sIgM in manually gated (as in Supporting Information Fig. S2) populations of B‐cell development (Fig. 3A) to their expression over the course of pseudotime in the trajectory (Fig. 3B). In agreement between manual analysis and pseudotime inference, we found the maximum level of nuclear iTdT in Pro‐B/PreB‐I stage, CD10 in PreB‐I stage and sIgM in Transitional B‐cells (Fig. 3B); however, the pseudotime plots showed single‐cell data with all gradual transitions. Thereafter, we examined the dynamics of expression of other markers in the early (stem cells to pre‐BII; Fig. 3C), mid (pre‐BII to transitionals; Fig. 3D), and late (transitionals to natural effectors; Fig. 3E) phases of B‐cell development. See Supporting Information Fig. S10 for a continuation of the expression to the switched memory subset.

Expression of iTdT in the pro‐B cells was followed by the CD127 (IL‐7Rα), CXCR4, iPAX‐5, iKi‐67, and CD10 expression at pro‐B to pre‐BI transition. Notably, CD127 rose and declined before iKi‐67 peaked in Pre‐BI while a second (smaller) CD127 peak followed by iKi‐67 was seen in pre‐BII, in line with the reported role of IL‐7 signaling inducing proliferation in humans [16]. Similarly, iPAX‐5 peak follows the CD127 peak (Fig. 3C). The iBcl‐2 and CD44 present bimodal expression, peaking at pro‐B stage first and again in mature stages in the peripheral blood (Fig. 3D). CD9 peaks within the Immature stage, followed by sIgM, while CD20 and IgD peak at transitional B‐cell stage (Fig. 3D). Finally, the CD22 and CXCR5 increase to their peaks at Naive and Effector stage (Fig. 3E), respectively, followed by CD73, which is down modulated in the natural effector cells and upregulated again in the switched memory B cells (Supporting Information Fig. S10C). CD27 is known as a B‐cell memory marker, but in fact, has also bi‐modal expression with a first peak at pre‐BI and pre‐BII stages and a second peak at memory stages. The expression of CD24 is first elevated in the pro‐B to the transitional stage only to reach its highest level in natural effectors (Fig. 3E).

**Figure 4:**
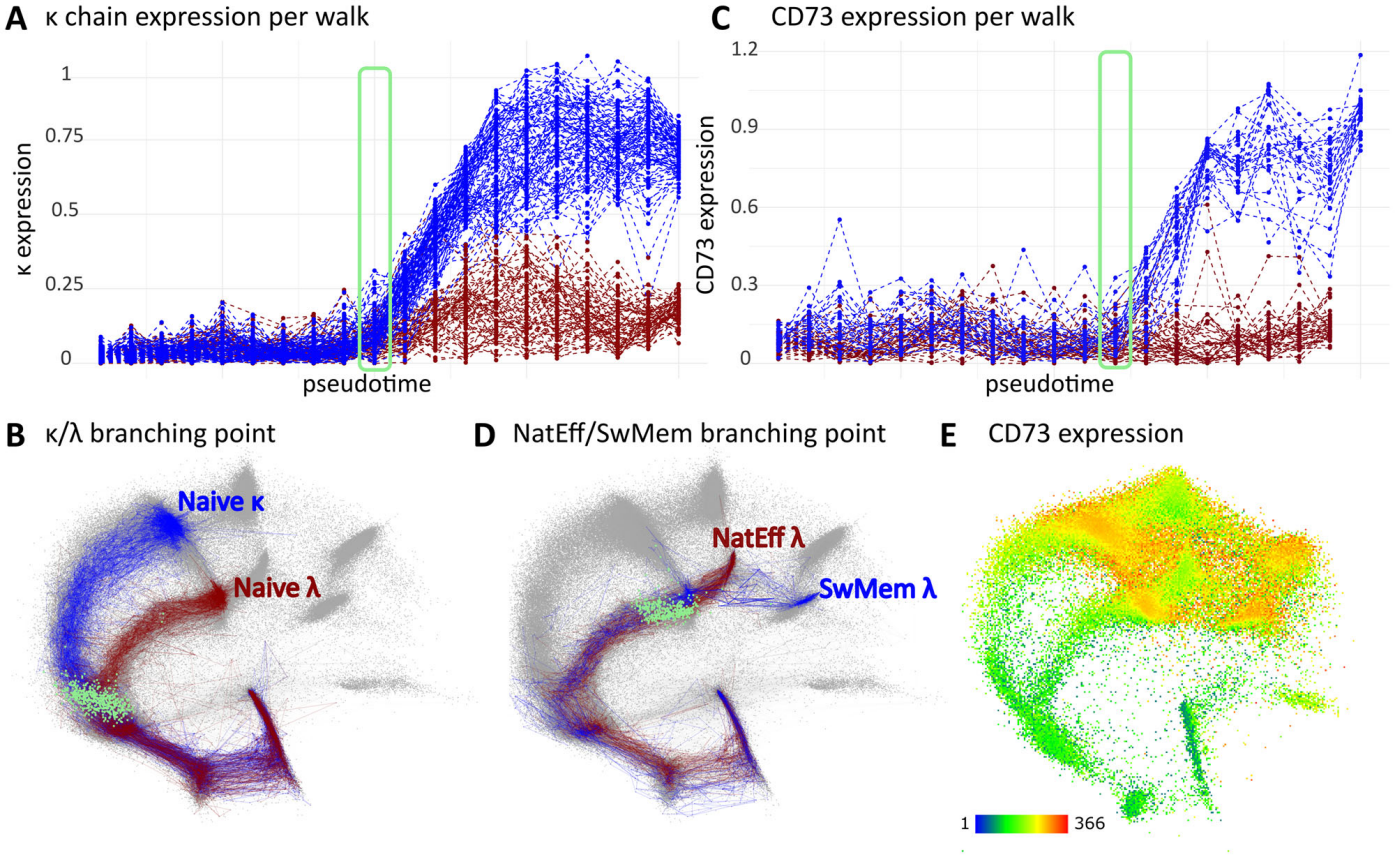
Identification of branching points in trajectories leading to the selected endpoints. (A) Pseudotime line plot showing expression of the κ chain for the selected trajectories leading to naive *κ* (blue) and naive *λ* (red) ends. The green rectangle indicates the branching point of trajectories and is projected as green dots on the *vaevictis* plot. (B) *Vaevictis* plot showing trajectories to naive *κ* (blue) and naïve *λ* (red) subsets with a projection of cells located in the branching point (green). (C) Pseudotime line plot showing the expression of CD73 for the selected trajectories leading to switched memory *λ* (blue) and natural effector *λ* (red) ends. The green rectangle indicates the branching point of trajectories and is projected as green dots in the *vaevictis* plot. (D) *Vaevictis* plot showing trajectories to natural effector *λ* (red) and switched memory *λ* (blue) endpoints with a projection of cells located in the branching point (green). (E) Expression of CD73 in the B‐cell compartment on the *vaevictis* plot.

Analyzing the two compartments separately, we could see that transitional, naive, and natural effector cells were present in both the bone marrow and the peripheral blood (Supporting Information Fig. S11). Their pseudotime position was slightly different suggesting there is a phenotypic difference. Indeed, we found higher expression of CXCR4 and lower expression of CXCR5 in the subsets in the bone marrow compartment (Supporting Information Fig. S11). The comparison of the Switched Memory subsets is shown in Supporting Information Fig. S12. To prove we can indeed find and describe the branching points of discovered trajectories we investigated trajectories to naive *κ* and *λ* endpoints against pseudotime (Fig. 4A). As expected, the branching point was located at the pre‐BII to Immature transition on a *vaevictis* projection (Fig. 4B). Analogously, we investigated the branching point in the of natural effector and switched memory cells (selected trajectories in Supporting Information Fig. S13). We found a branching point at a trajectory segment where CD73 started to increase on the way to the switched memory B cells (Fig. 4C). The branching point was topologically located at the transitional to naïve B‐cells (Fig. 4D). We identified two trajectories leading up to natural effector B‐cell endpoint, one devoid of CD73 expression and a second with only transient increase of CD73 expression (Supporting Information Fig. S14). The expression of CD73 is variable in the peripheral blood B‐cell compartment, with low expression in the natural effectors (Fig. 4E), however in the investigated trajectories it was preceded by CXCR5, a germinal center homing marker (Supporting Information Fig. S14D). In the trajectories to the plasma cells, we noticed both: natural effectors skipping and natural effectors passing trajectories, where the passing trajectories exhibited heterogeneous CD73 expression with shifted kinetics (Supporting Information Fig. S15). Taken together, the identification of branching points in logical developmental stages together with the context of markers changes proved that *tviblindi* can be used to properly describe B‐cell development.

**Figure 5:**
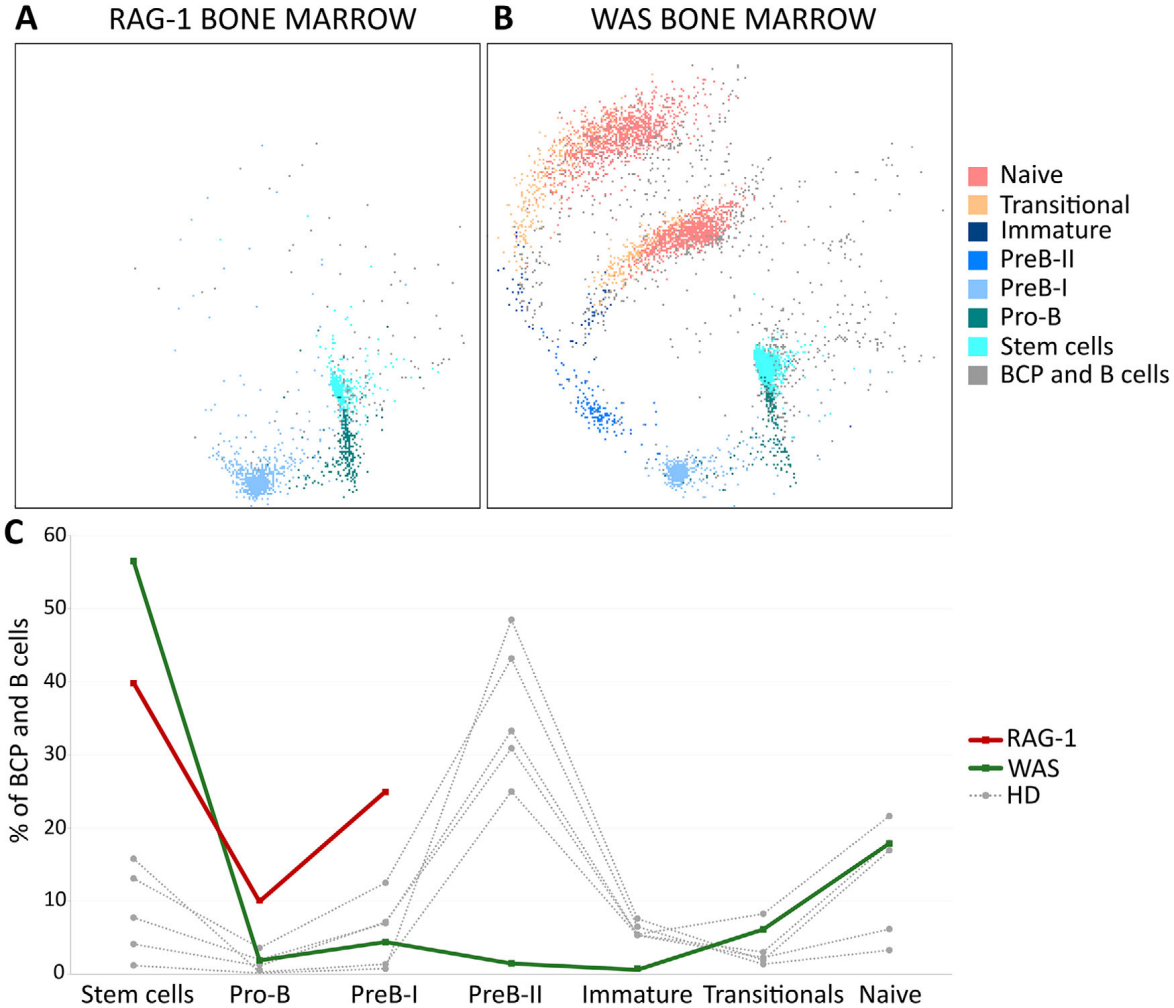
RAG‐1 and WAS patients’ bone marrow B‐cell compartment. (A) RAG‐1 patient and (B) WAS patient with an overlay of manual gates on the *vaevictis* plot. (C) Relative distribution of B‐cell subsets across the bone marrow B‐cell precursor and B‐cell developmental stages in patients compared with five healthy donors.

Finally, we investigated abnormal and abrupt B‐cell development in the bone marrow in patients with known PIDD‐causing mutations. As expected, the mutation in the RAG‐1 gene caused a developmental block at the PreB‐I stage of development (Fig. 5A). The WAS patient bone marrow showed a high proportion of CD34^+^ stem cells (initially raising suspicion of malignancy) and a complete lack of expansion at the PreB‐II stage (although iKi67 was as high as in HD). Additionally, we observed a decrease in the immature cells in the WAS bone marrow (Fig. 5B and C). In the peripheral blood, the WAS patient showed a higher proportion of stem and transitional cells and a lack of natural effector, switched memory, and plasma cells Supporting Information Fig. S16). By selecting the full developmental trajectories of healthy donors, RAG‐1, and WAS patients, we were able to superimpose them and display the marker expression versus pseudotime. As expected, the RAG‐1 patient presented with high, albeit dropping iTdT together with decreasing iBcl‐2 at the PreB‐I stage prior to the developmental block. The WAS patient's bone marrow showed a similar expression pattern for these markers. Interestingly, we observed asynchronously higher expression of iCD79a in both the RAG‐1 and WAS patients at the PreB‐I and PreB‐II stages but not in mature B‐cell stages (Supporting Information Fig. S17).

## Conclusion

We presented a single‐cell analysis solution for interrogation of early B‐cell developmental trajectories on multiple samples of relevant tissues (bone marrow and peripheral blood). We designed and validated a mass cytometry panel capable of evaluating 30 markers plus 5 sample barcodes. We compared its performance to a benchmark of EuroFlow 10‐color cytometry assay. We showed a practical, feasible, and scalable *vaevictis* projection calculation based on deep learning.

This tool is designed to create a continuous representation of the data rather than isolated clusters allowing a clear interpretation of the dynamics in the data (as compared with other currently used methods t‐SNE [17] or UMAP [18]). Due to the naïve importance sampling, numerically dominant populations are not overrepresented in the 2D plot and the running time is basically insensitive to the size of the original dataset. The deep learning architecture allows for direct reuse of the trained representation on a newly acquired sample (if performed in a standardized manner as in this study the additional HD donors’ and PIDD patients’ bone marrows were mapped to the same *vaevictis* projection as the healthy bone marrow samples measured and analyzed several months earlier).

Thus, uniquely, samples of different donors (affected and unaffected) and multiple tissues (central and peripheral) can be probed with thousands of putative pathways, that are defined by a starting point, markers used, and the overall definition of cells belonging to the pool of relevant cell type (here stem cells and B‐cell lineage). User‐defined terminal points can be added if focused analysis is desired.

All trajectories found are visually presented for interrogation, diverse terminal ends can be selected and individual trajectories are assembled into relevant pathways for further exploration. Recent mathematical apparatus based on persistence homology calculations is used to quantitatively describe similarities of trajectories that can be assembled together.

Notably, trajectories found in our dataset correspond to the known theory of B‐cell development, they logically transit from the central organ of hematopoiesis (bone marrow) to the periphery (blood). When dissected in detail, they show expected sequences of canonical markers but add detail to the transition points and provide dynamic information about the expression of markers within known stages. For example, CD127 peaking before PAX‐5 is in line with a recent study showing an important role of CD127 (IL‐7RA) signaling in promoting PAX‐5 expression [16]. While CD27 is conventionally used for the phenotypic description of memory B‐cell subsets in the periphery, we show its upregulation also in the pre‐B stages, as shown earlier by Vaskova et al. [19]. The transient downregulation of CXCR4 and simultaneous CD9 upregulation which we see within the pre‐BI stage is in line with Leung et al. [20], who observed that CD9 levels are enhanced after SDF‐1 stimulation suggesting that CD9 plays a role in the SDF‐1/CXCR4 axis known to be essential in HSC/progenitor homing. The expression profile of CD24 along the calculated pseudotime follows the experimental findings of studies [21, 22] showing the highest peak of expression in transitional B cells (followed by a decrease in naive cells) and the second in memory B cells. While the mature B‐cell stages were immunophenotypically similar in the bone marrow and peripheral blood (found in the same regions on *vaevictis* plot), we could find quantitative differences in the CXCR4 and CXCR5 expression, known homing receptors [23, 24]. Our approach allowed us to investigate multiple developmental endpoints resulting from trajectories’ branching. The CD73, a known marker of switched memory B cells [25] gradually increased until the switched memory B‐cell stage, while it remained negative or only transiently increased toward the natural effector B‐cell stage. While the branching point was found at the naïve B‐cell stage, the heterogenous expression of CD73 together with CXCR5 expression suggested that there are still alternative trajectories among the natural effector B cells. One extrafollicular trajectory seems defined by the absence of CXCR5 and CD73 (CXCR5 and CD73 negative), while a second trajectory, defined by a transient expression of CXCR5, may describe cells that passage transiently in the germinal center. Indeed, the distinction between extrafollicular B cells and natural effector‐B cells is still unclear [26], and our analysis can provide insights into marker definition to dissect distinct cell fates. Surprisingly, the pathways that pass through the natural effector stage en route to plasma cells express CD73 at different levels (lower levels or not at all). However, CD73 expression only peaks after CD27 acquisition, as opposed to CD73 peaking before CD27 acquisition in the switched memory B‐cell stage. In addition, we have previously reported the performance of the *tviblindi* algorithm on an artificial dataset with multiple branchings and used it to describe the thymocyte development in the thymus [15].

Unlike the so far published algorithms that oversimplify the trajectory inference showing a single dominant trajectory or two trajectories with a single branching point (e.g. Wishbone [27]), we could analyze multiple branching points and bring quantitative expression information as well as topological information about the branching point. The theoretical limitation is the number of investigated markers and the choice of tissues and samples. This can be overcome by using *tviblindi* on a single‐cell RNASeq or better yet CITE‐Seq dataset combining the protein markers with gene expression and enriching the mass cytometry panel in the next iteration.

We can generalize, that *tviblindi* algorithm can reliably show the sequence of expression of surface markers as well as cytoplasmic markers and nuclear transcription factors. We also show that our mass cytometry panel and *tviblindi* could be used to compare healthy reference with affected (RAG‐1 and WAS patients) development to discover alterations in the development. While the RAG‐1 patient presented the expected block at the PreB‐I stage, we could also observe the kinetics of markers before the block (decreasing iBcl‐2, iTdT, and iCD79a). Defective WAS protein affects motility, BCR‐signaling, and class switch in B cells [28]. Similar to our patient, clinical suspicion for juvenile myelomonocytic leukemia has been reported as a presenting symptom of the WAS [29]. Numerical abnormalities in the bone marrow B‐cell subsets were reported before by Castiello et al. [30], where decreased immature B cells were reported. The stages of bone marrow B‐cell development where WAS patient shows failure to expand positively selected cells are stages with low antiapoptotic protein iBcl‐2, opening questions of WAS protein role in tuning antiapoptotic signaling in developing B cells since increased apoptosis was described in human WAS patients’ peripheral lymphocytes [31].

These detailed developmental deviations found by *tviblindi* could potentially disclose targetable processes for therapy of PIDD and/or other diseases (such as B‐cell neoplasia).

## Conflict of interest

The authors declare no financial or commercial conflict of interest.

**Abbreviations**
GUI: graphical user interface
PIDD: primary immunodeficiency disorders
TI: trajectory inference
WAS: Wiskott–Aldrich syndrome

## Supporting information

SUPPORTING INFORMATION

SUPPORTING INFORMATION

## Data Availability

The data that support the findings of this study are openly available in the GitHub repository at https://github.com/tomas‐kalina/B‐celldevelopmentFCS.
