# Supplementary material for: Tviblindi algorithm identifies branching developmental trajectories of human B‐cell development and describes abnormalities in RAG‐1 and WAS patients
Source: Eur J Immunol. 2024 Sep 5;54(12):2451004. doi: 10.1002/eji.202451004 (PMC11628918; doi:10.1002/eji.202451004)
Supplement: Supplementary file 1 — SUPPORTING INFORMATION [file EJI-54-2451004-s002.pdf]

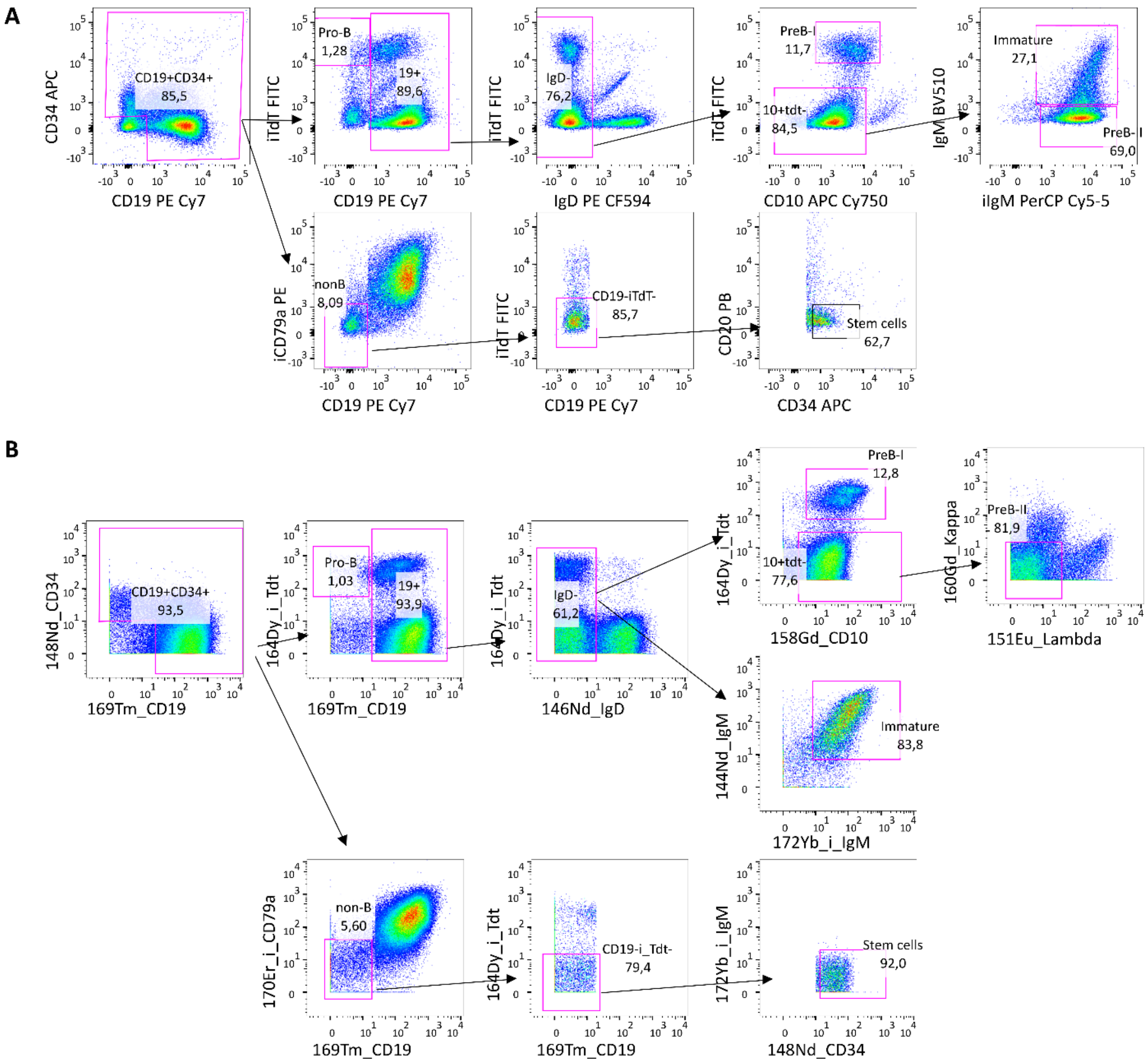

Supplementary Figure 1. Gating strategy for (A) flow cytometry and (B) CyTOF data. Representative results from the same healthy bone marrow sample measured in parallel.

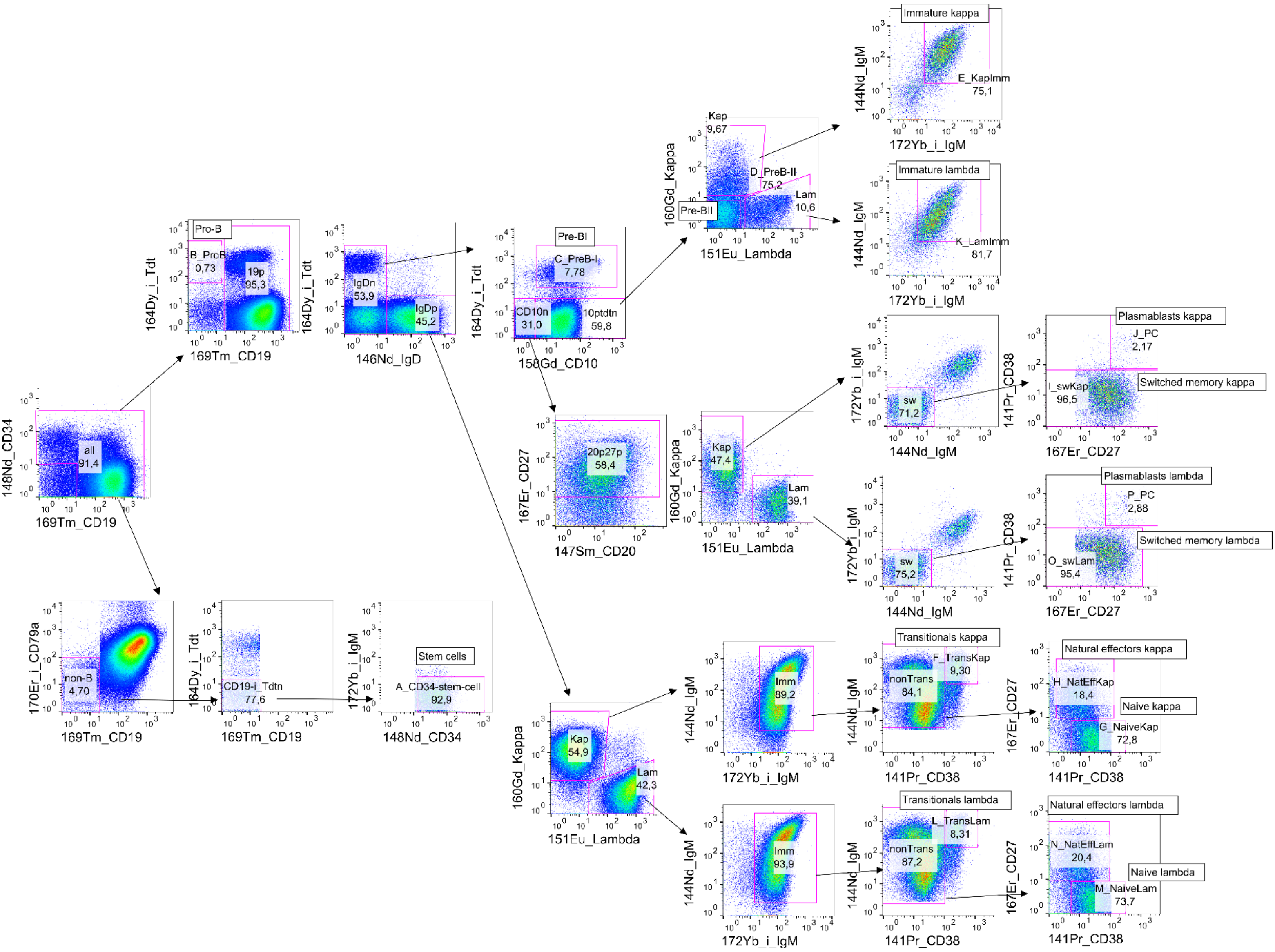

Supplementary figure 2. Gating strategy for the CyTOF data from healthy donor bone marrow and peripheral blood samples.

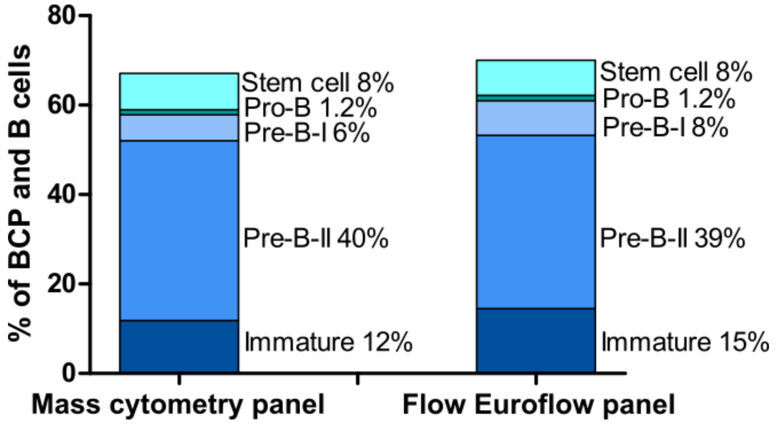

Supplementary Figure 3. Distribution of B-cell precursor populations across the novel mass cytometry panel (left) and previously validated flow Euroflow panel (right). Colors indicate individual populations with percentage representation within both of the panels. Stem cells were defined as CD19-CD79α-TdT-CD34+, Pro-B cells as CD19-TdT+CD34+, Pre-BI cells as CD19+TdT+CD34+, Pre-BII cells as CD19+CD10+iIgM+IgM- and Immature cells as CD19+CD10+iIgM+IgM+. Results from 4 different healthy bone marrow samples measured in parallel.

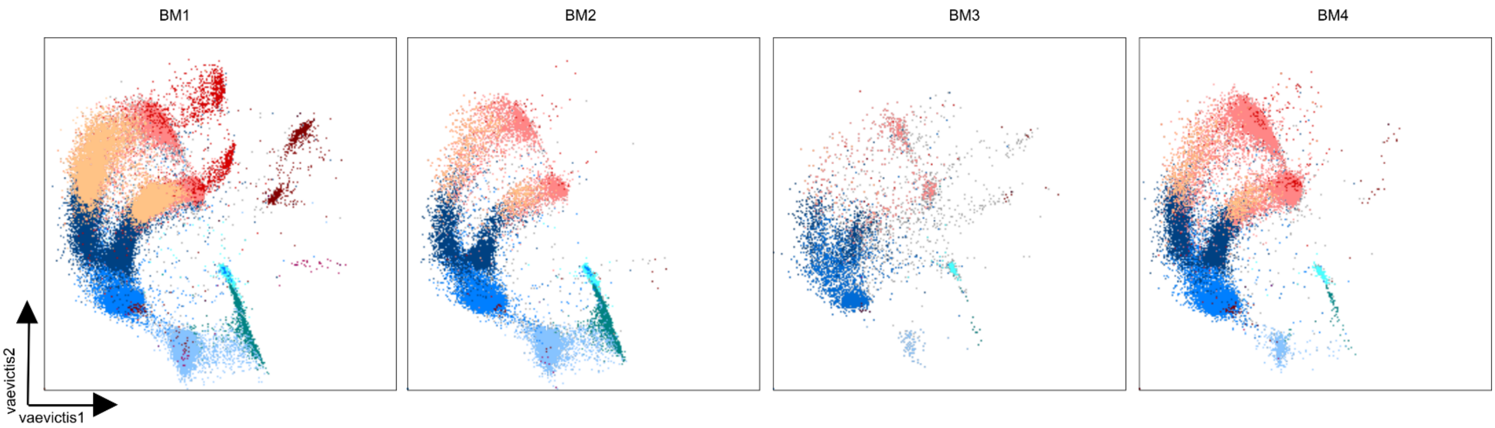

Subset Name	Count
Plasmablasts	55,0
Switched memory	713
Natural Effector	1764
Naive	41570
Transitionals	9364
Immature	9817
PreB-II	46390
PreB-I	9345
Pro-B	1327
Stem cells	4825
BM1	135926

Subset Name	Count
Plasmablasts	8,00
Switched memory	24,0
Natural Effector	85,0
Naive	3328
Transitionals	762
Immature	3520
PreB-II	24082
PreB-I	6513
Pro-B	1610
Stem cells	6218
BM2	59672

Subset Name	Count
Plasmablasts	1,00
Switched memory	19,0
Natural Effector	24,0
Naive	558
Transitionals	30,0
Immature	662
PreB-II	2132
PreB-I	83,0
Pro-B	16,0
Stem cells	839
BM3	6803

Subset Name	Count
Plasmablasts	5,00
Switched memory	71,0
Natural Effector	305
Naive	14231
Transitionals	1001
Immature	5502
PreB-II	37954
PreB-I	536
Pro-B	81,0
Stem cells	672
BM4	69935

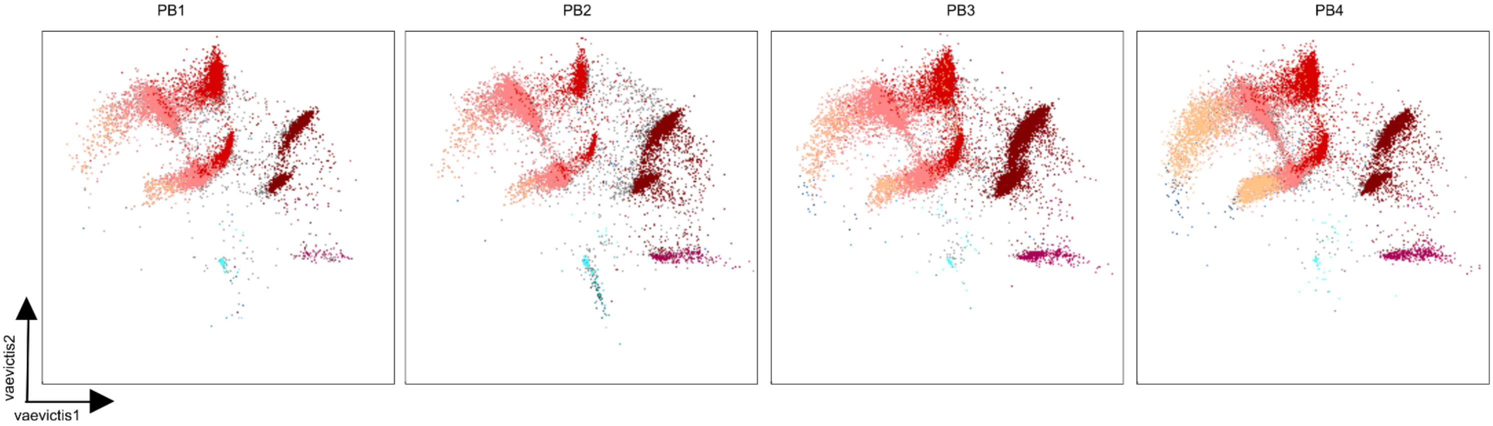

Subset Name	Count
Plasmablasts	69,0
Switched memory	2290
Natural Effector	3445
Naive	7955
Transitionals	327
Immature	6,00
PreB-II	7,00
PreB-I	5,00
Pro-B	6,00
Stem cells	46,0
PB1	19000

Subset Name	Count
Plasmablasts	231
Switched memory	4518
Natural Effector	1368
Naive	9624
Transitionals	234
Immature	11,0
PreB-II	31,0
PreB-I	4,00
Pro-B	42,0
Stem cell	110
PB2	23429

Subset Name	Count
Plasmablasts	448
Switched memory	14881
Natural Effector	18903
Naive	10895
Transitionals	734
Immature	81,0
PreB-II	67,0
PreB-I	2,00
Pro-B	1,00
Stem cells	53,0
PB3	59372

Subset Name	Count
Plasmablasts	422
Switched memory	5823
Natural Effector	5317
Naive	48406
Transitionals	2556
Immature1	75,0
PreB-II	32,0
PreB-I	3,00
Pro-B	3,00
Stem cells	47,0
PB4	81686

Supplementary Figure 4. *Vaevictis* plots of bone marrow and peripheral blood for the individual samples. Visualization of the five healthy donor bone marrow samples (top row, BM1-BM4) and the five healthy donor peripheral blood samples (bottom row, PB1-PB4) with manually gated populations applied to the graph in color, with annotation and counts of the individual subsets.

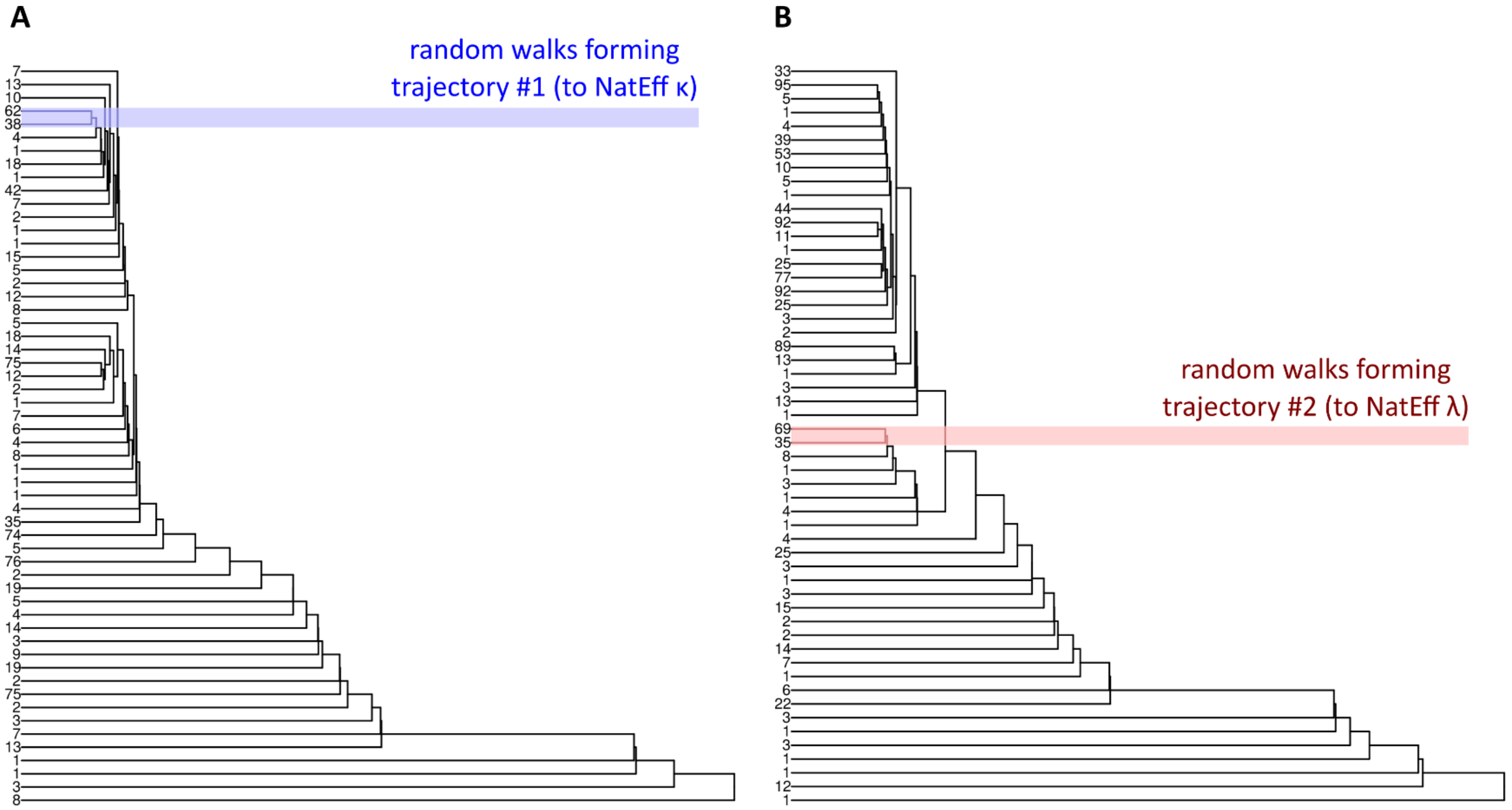

Supplementary Figure 5. Hierarchical clustering dendrogram for Natural Effector κ and λ developmental trajectories. Dendrograms show clustering of all random walks leading to developmental endpoints located in the (A) Natural Effector κ and (B) Natural Effector λ clusters. Leaves represent the groups of random walks with similar topology. In blue is highlighted the selected group of walks representing developmental trajectory to Natural Effector κ cells. In red is highlighted the group of walks representing developmental trajectory to Natural Effector λ cells.

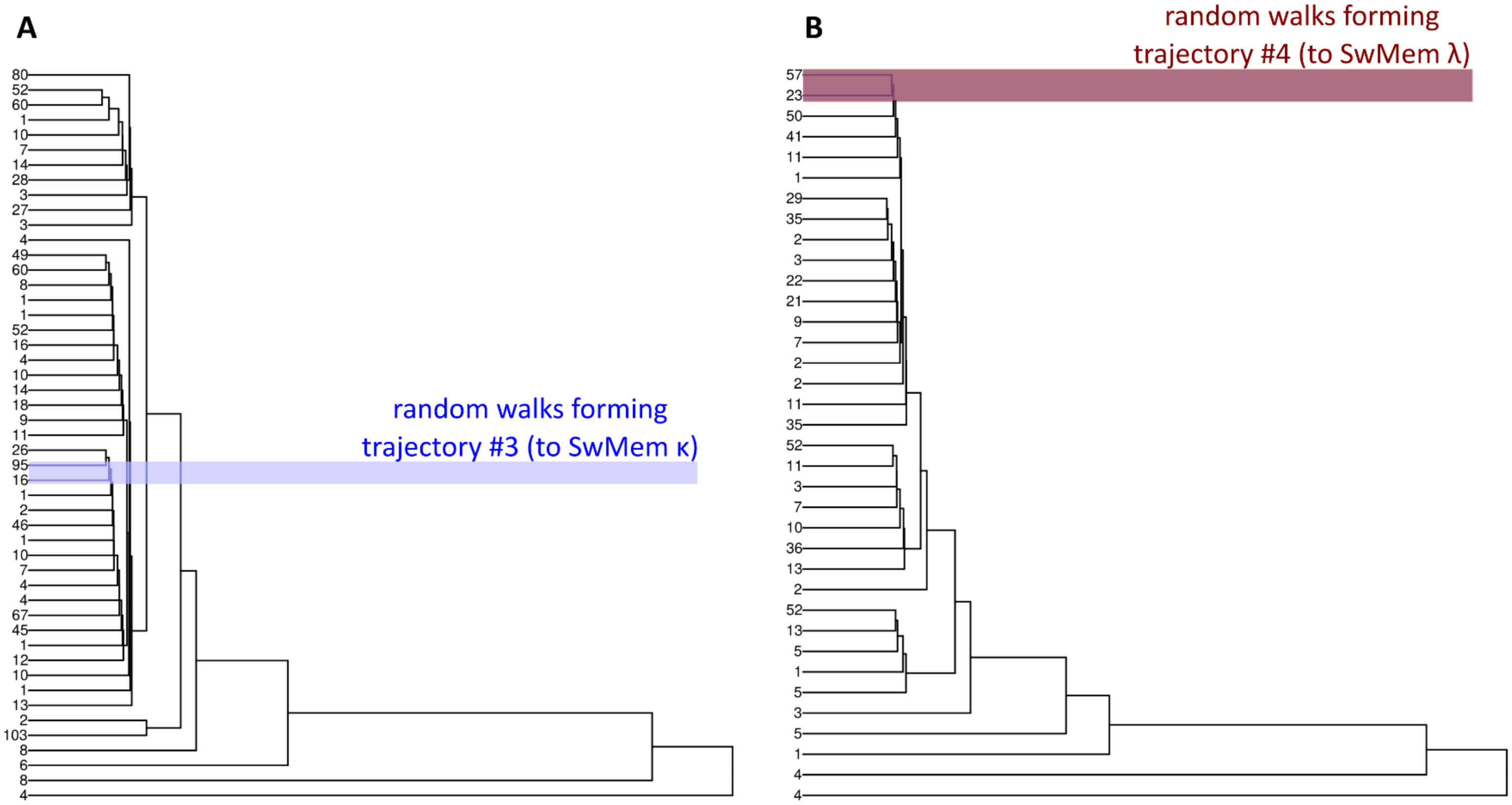

Supplementary Figure 6. Hierarchical clustering dendrogram for Switched Memory κ and λ developmental trajectories. Dendrograms show clustering of all random walks leading to developmental endpoints located in the (A) Switched Memory κ and (B) Switched Memory λ clusters. Leaves represent the random walks and their abundance. In blue is highlighted the selected group of walks representing developmental trajectory to (A) Switched Memory κ cells and (B) Switched Memory λ cells.

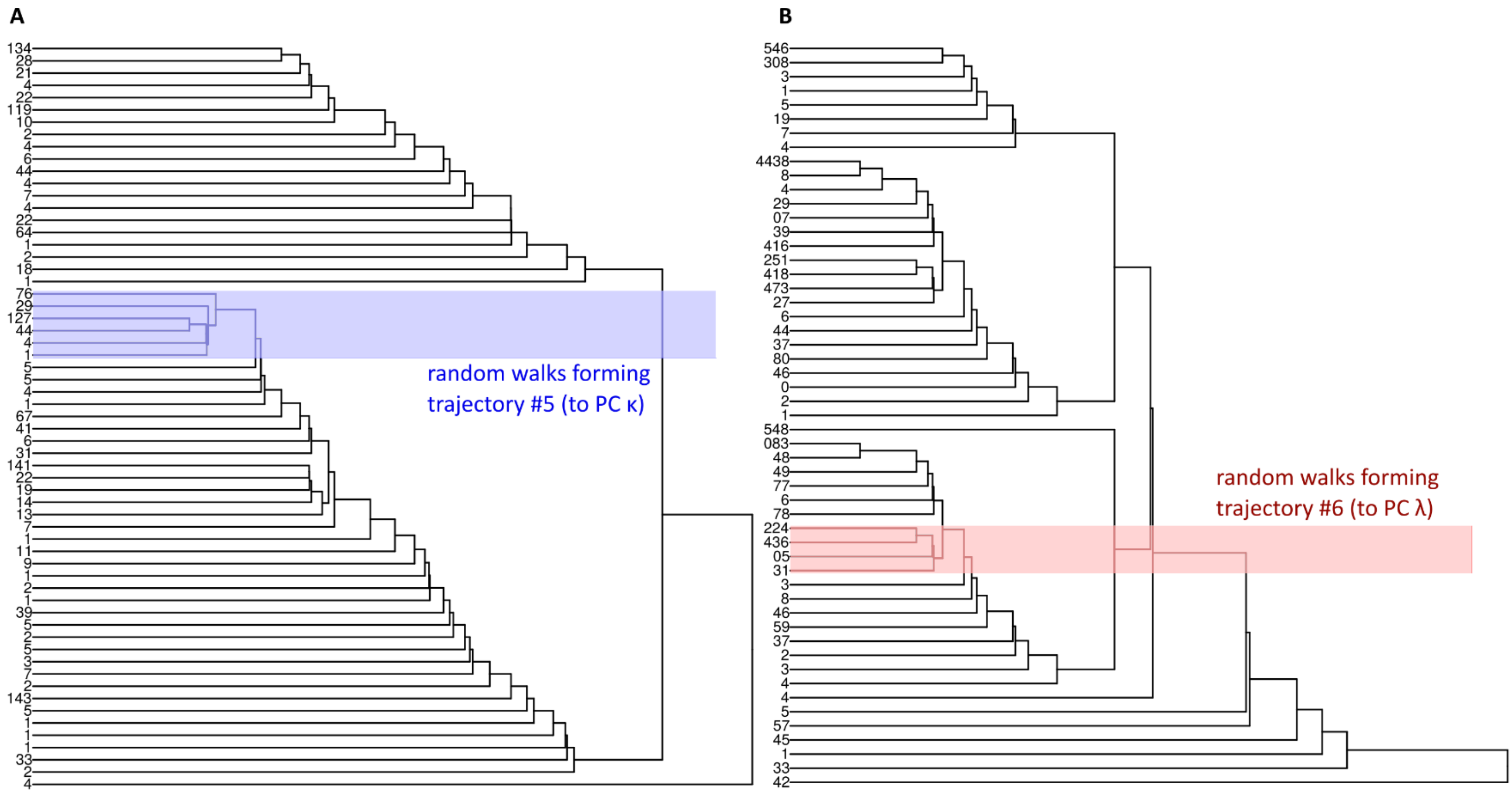

Supplemetary Figure 7. Hierarchical clustering dendrogram for Plasma cells (PC) κ and λ developmental trajectories. Dendrograms show clustering of all random walks leading to expert added developmental endpoints located in the (A) PC κ and (B) PC λ clusters. Leaves represent the random walks and their abundance. In blue is highlighted the selected group of walks representing developmental trajectory to (A) PC κ cells and (B) PC λ cells.

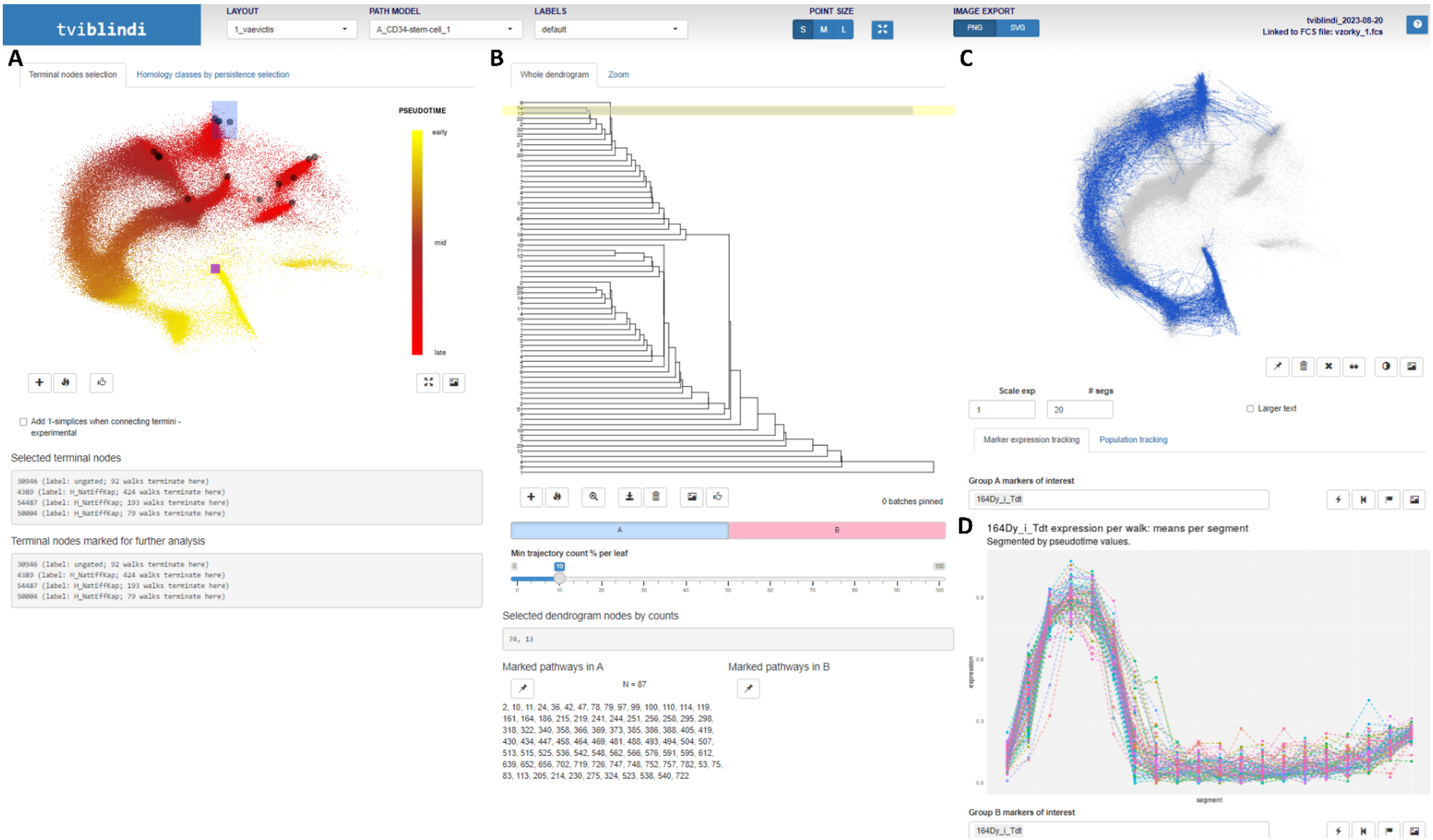

Supplementary Figure 8. *tviblindi* graphical user interface (GUI). (A) *vaevictis* plot with selected terminal endpoints located in the Natural Effector κ cluster, (B) hierarchical clustering dendrogram with selection of random walks forming the trajectory leading to Natural Effector κ cluster, (C) *vaevictis* plot with random walks selected in (B), (D) lineplot with the expression of iTdT along pseudotime of the selected trajectory.

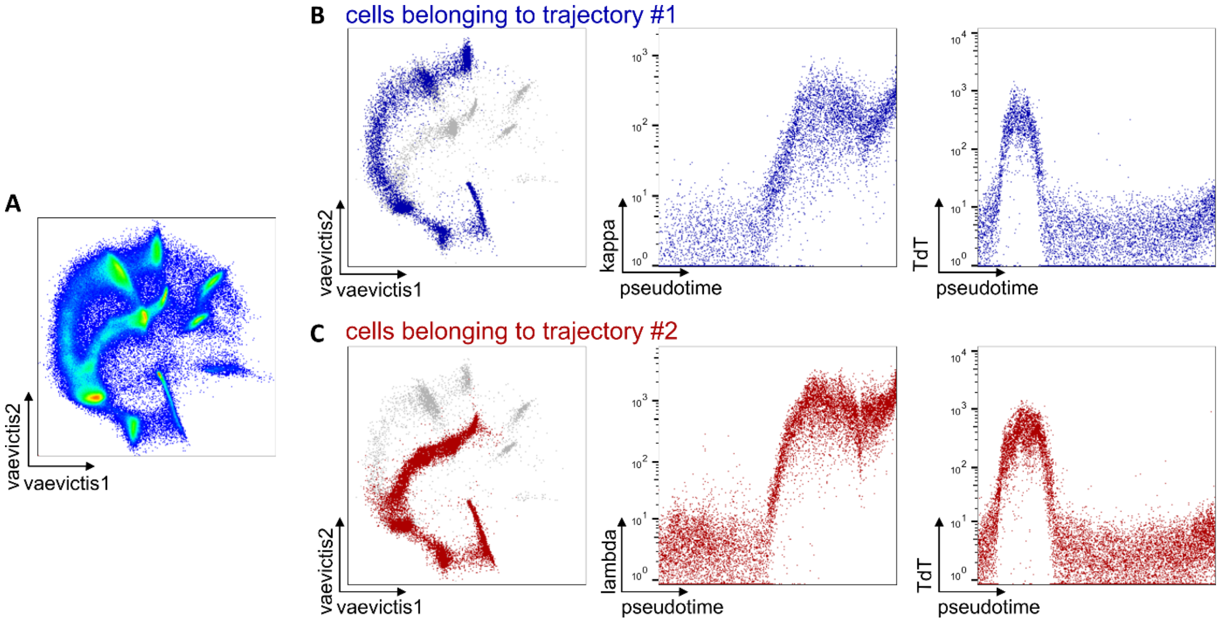

Supplementary Figure 9. Manual analysis of trajectories constructed by *tviblindi* using enhanced FCS file. *Vaevictis* plot of (A) the entire data set and (B, C) with colored cells belonging to the selected trajectory ending at the Natural Effector κ (blue) and λ (red) subsets in FlowJo. Expression of κ, λ and TdT markers along pseudotime.

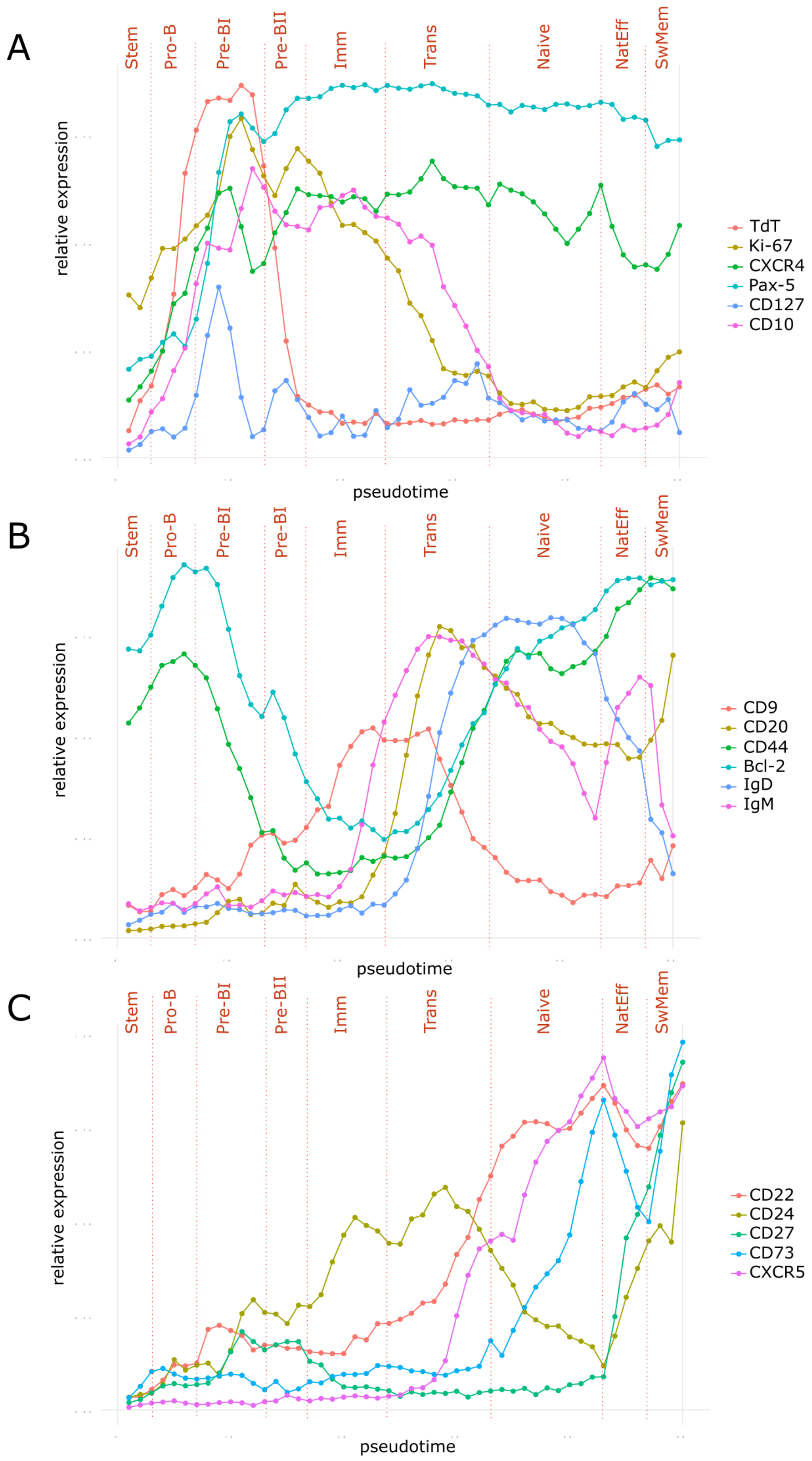

Supplementary Figure 10. Detailed analysis of the trajectory leading to Switched memory κ cells. Pseudotime line plots showing the average expression of markers upregulated in the (A) early, (B) mid and (C) late phase of the development.

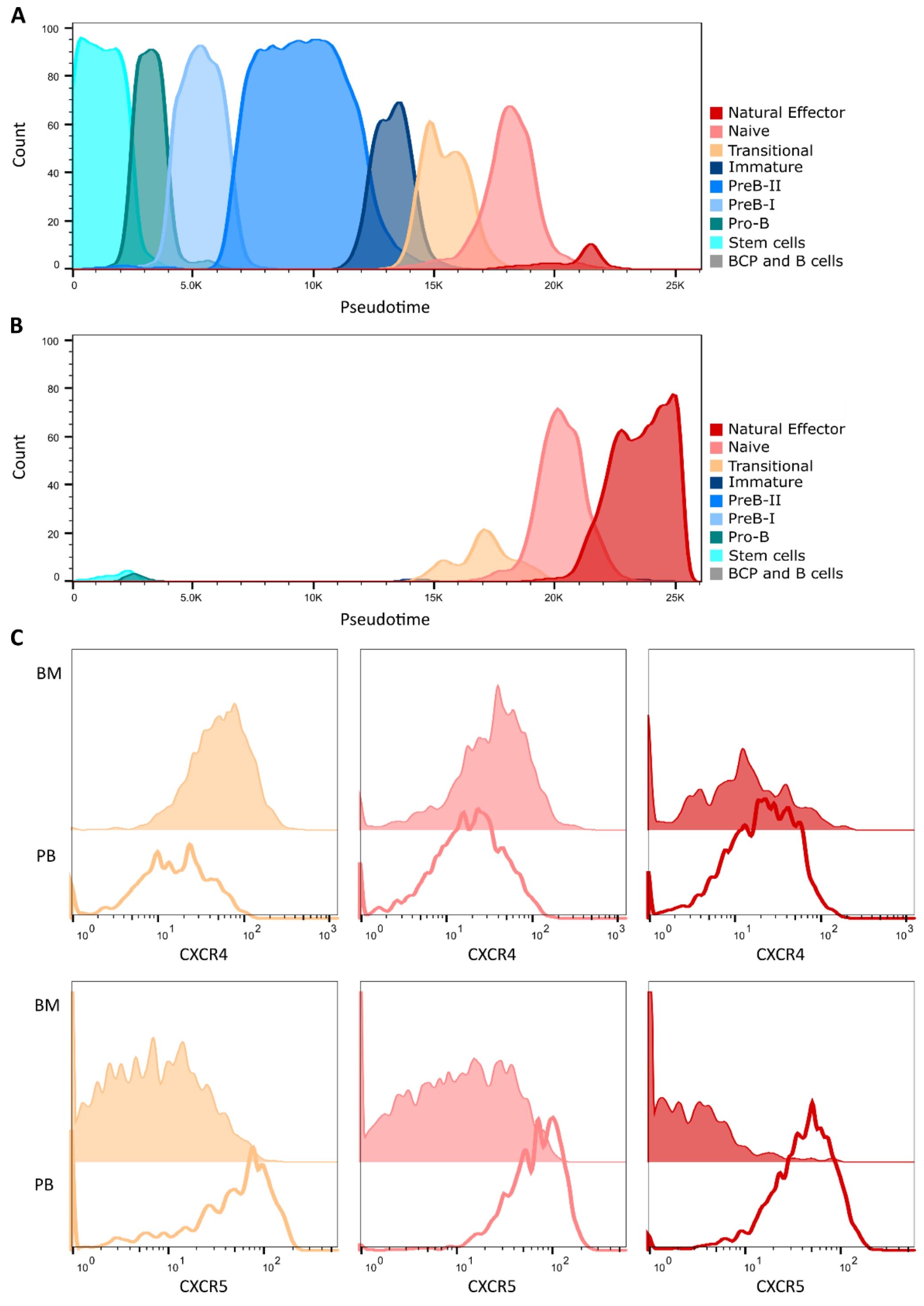

Supplementary Figure 11. Contribution of the B cell precursor (BCP) and B-cell stages to the bone marrow and peripheral blood compartments. Individual populations are shown as histograms in the data set divided into (A) bone marrow (n=4, concatenated) and (B) peripheral blood (n=4, concatenated). (C) Differential expression of the markers CXCR4 (top row) and CXCR5 (bottom row) in the populations which are present in both of the compartments. Solid line histograms indicate subsets present in the peripheral blood (PB). Filled histograms indicate subsets present in the bone marrow (BM).

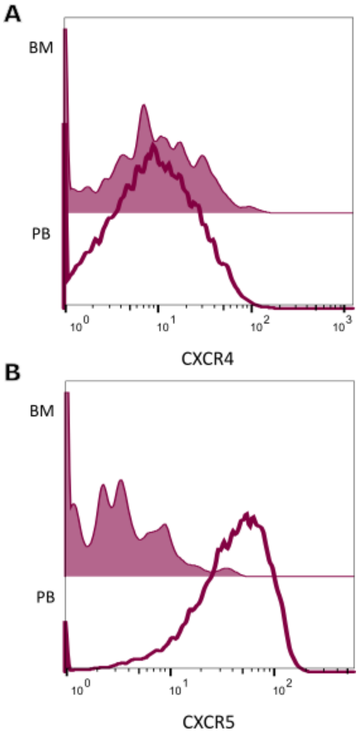

Supplementary Figure 12. Differential expression of the markers (A) CXCR4 and (B) CXCR5 in Switched memory cells present in both bone marrow (BM) and peripheral blood (PB) compartments. Solid line histograms indicate subsets present in the PB. Filled histograms indicate subsets present in the BM.

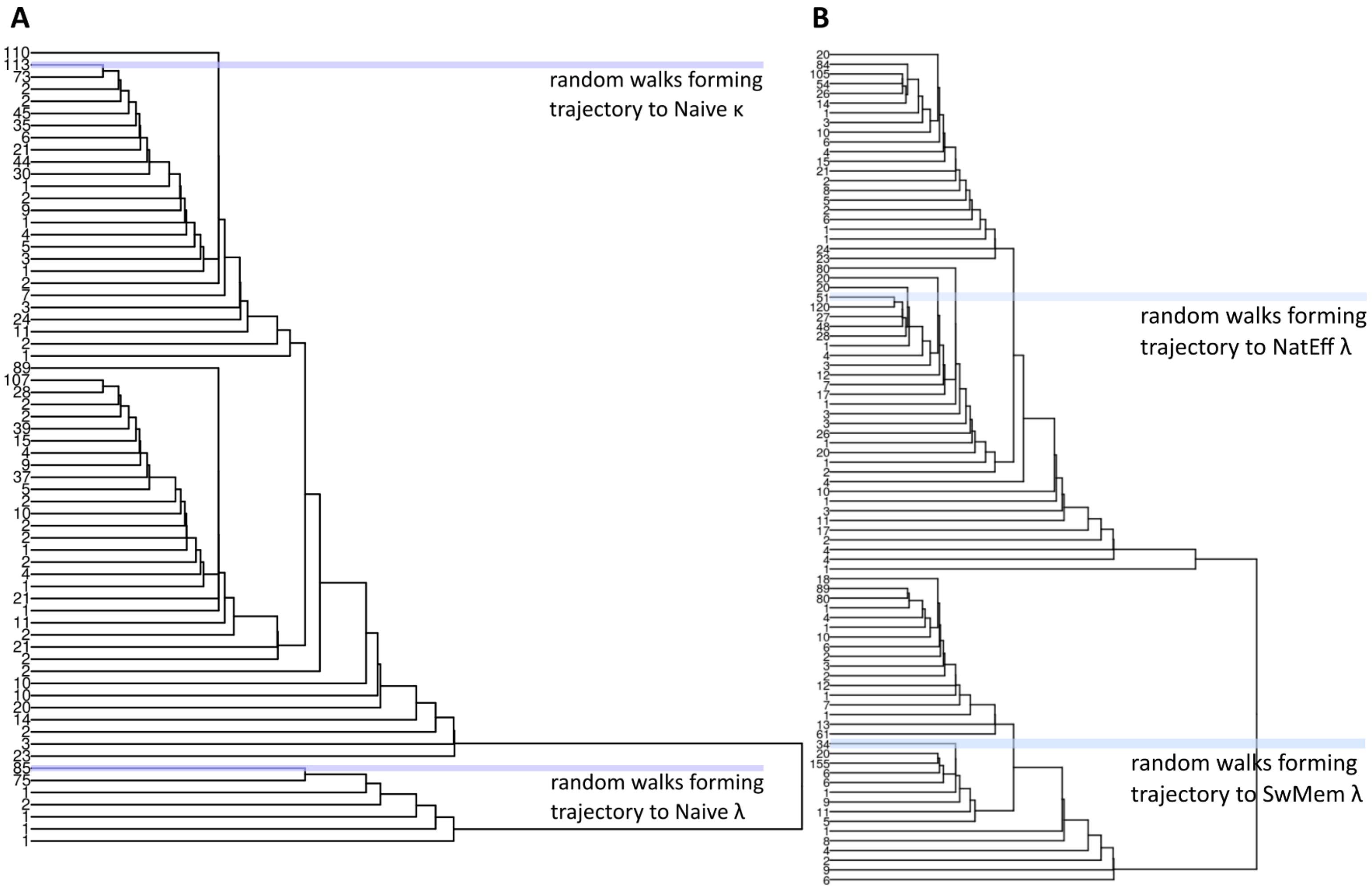

Supplementary Figure 13. Hierarchical clustering dendrograms. For the developmental branching of (A) Naive κ and λ cells and (B) Natural Effector λ and Switched memory λ cells. In blue are highlighted the groups of random walks representing the developmental trajectories to (A) Naive κ and λ cells and (B) Natural Effector λ and Switched memory λ cells.

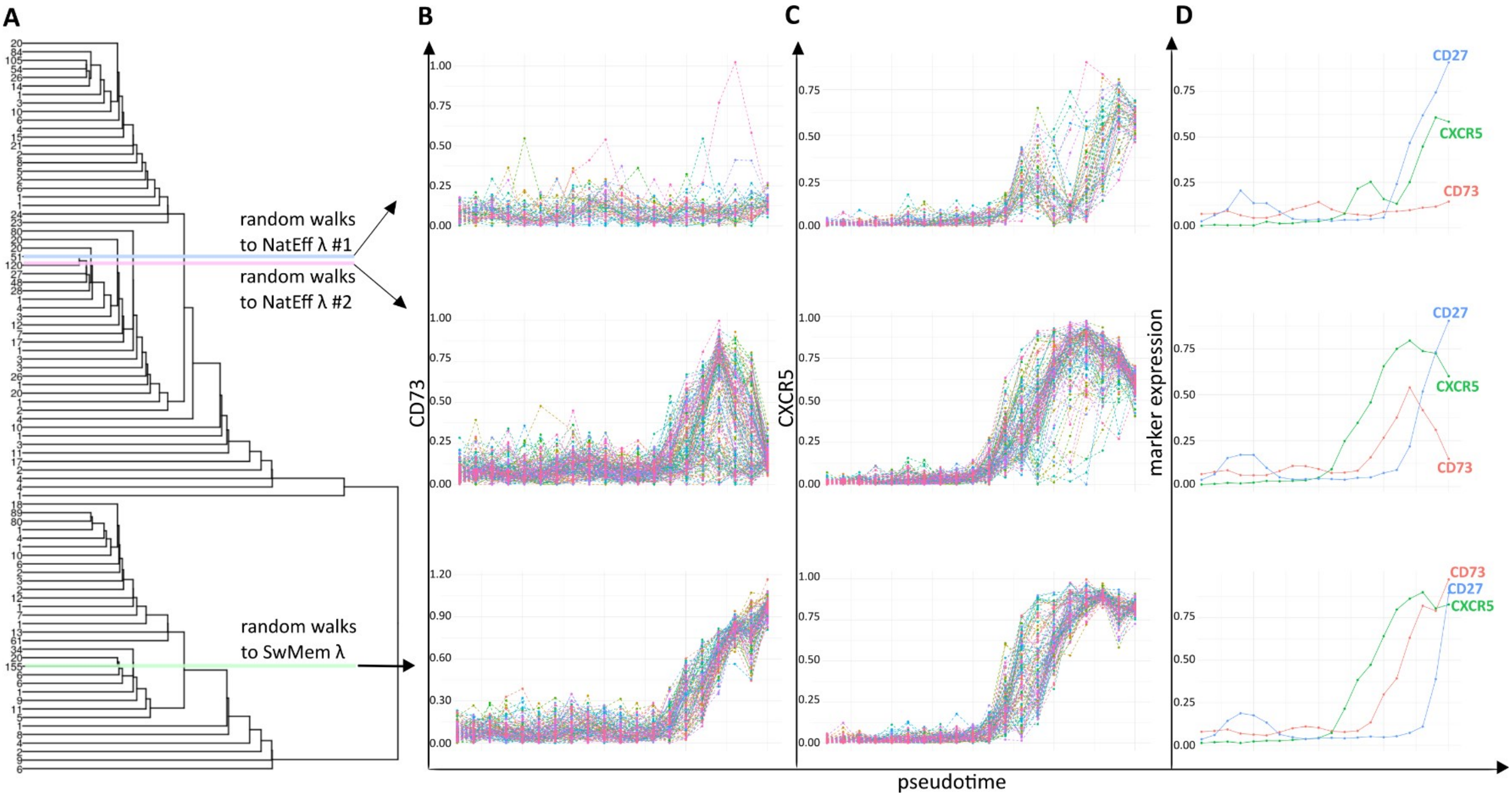

Supplementary Figure 14. Differential expression of the markers CD73 and CXCR5 in trajectories leading to Natural Effector and Switched memory cells. (A) Dendrogram with selection of random walks forming the trajectory to Natural Effector λ #1(blue), #2 (pink) and Switched memory λ (green). The trajectory to Natural Effector λ #1 shows no upregulation of CD73 (B, top) and transient expression of CXCR5 (C, top). The trajectory to Natural Effector λ #2 (B, mid) shows heterogeneous expression of CD73 and upregulation of CXCR5 (C, mid). The trajectory to Switched memory λ shows clear upregulation of both CD73 (B, bottom) and CXCR5 (C, bottom). (D) Lineplots for the different trajectories with dynamics of expression of CD73, CXCR5 and CD27.

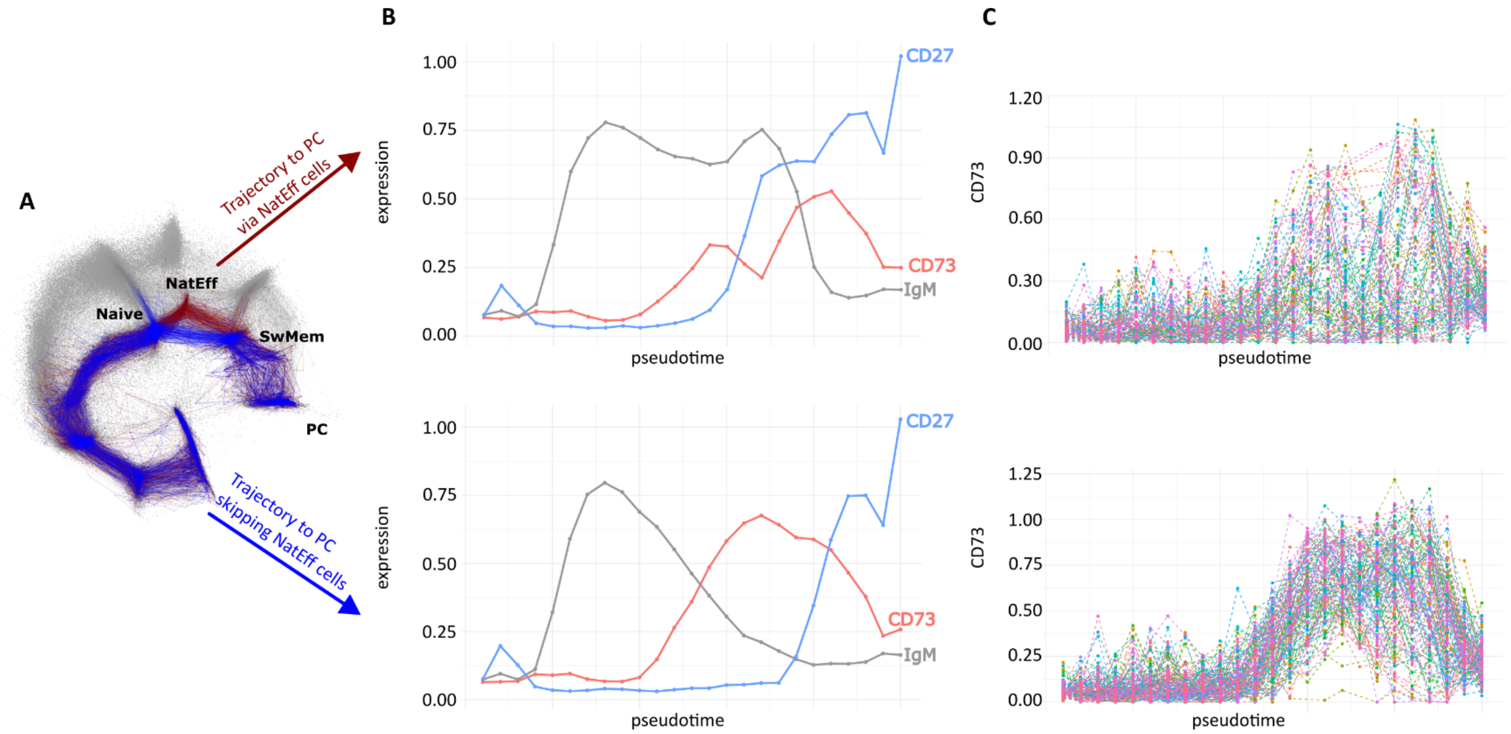

Supplementary Figure 15. (A) *Vaevictis* plot with displayed trajectories to Plasma cells (PC) passing through (in red) or skipping (in blue) Natural Effectors. (B) Lineplots showing the relative expression of the markers IgM, CD73 and CD27 along the pseudotime of the trajectory passing through (top) or skipping (bottom) Natural Effectors. (C) Relative expression of CD73 along the pseudotime of all walks forming trajectory to PC passing through (top) or skipping (bottom) Natural Effectors.

A

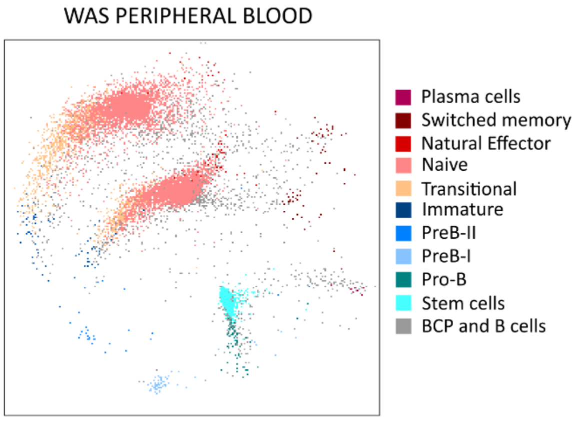

B

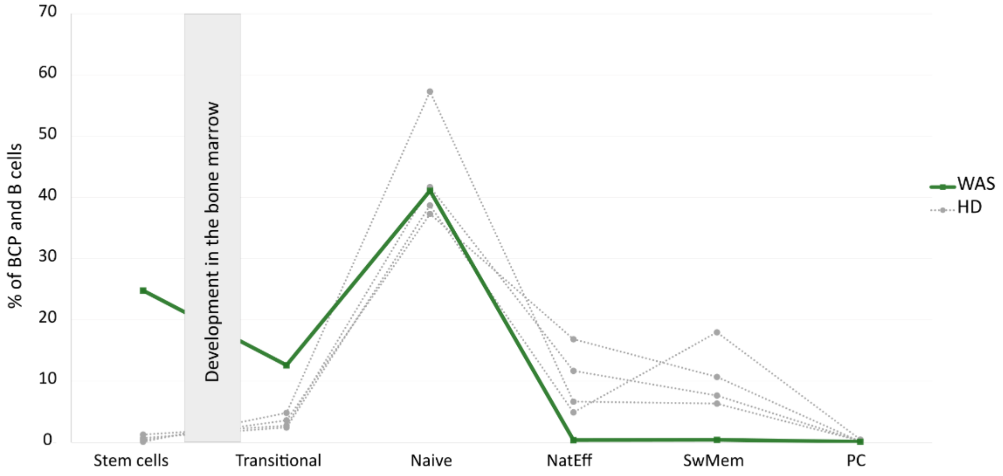

Supplementary Figure 16. WAS patient peripheral blood B cell compartment. (A) WAS patient with an overlay of manual gates on the *vaevictis* plot. (B) Relative distribution of B cell subsets across the peripheral blood developmental stages in WAS patient compared to 4 healthy donors.

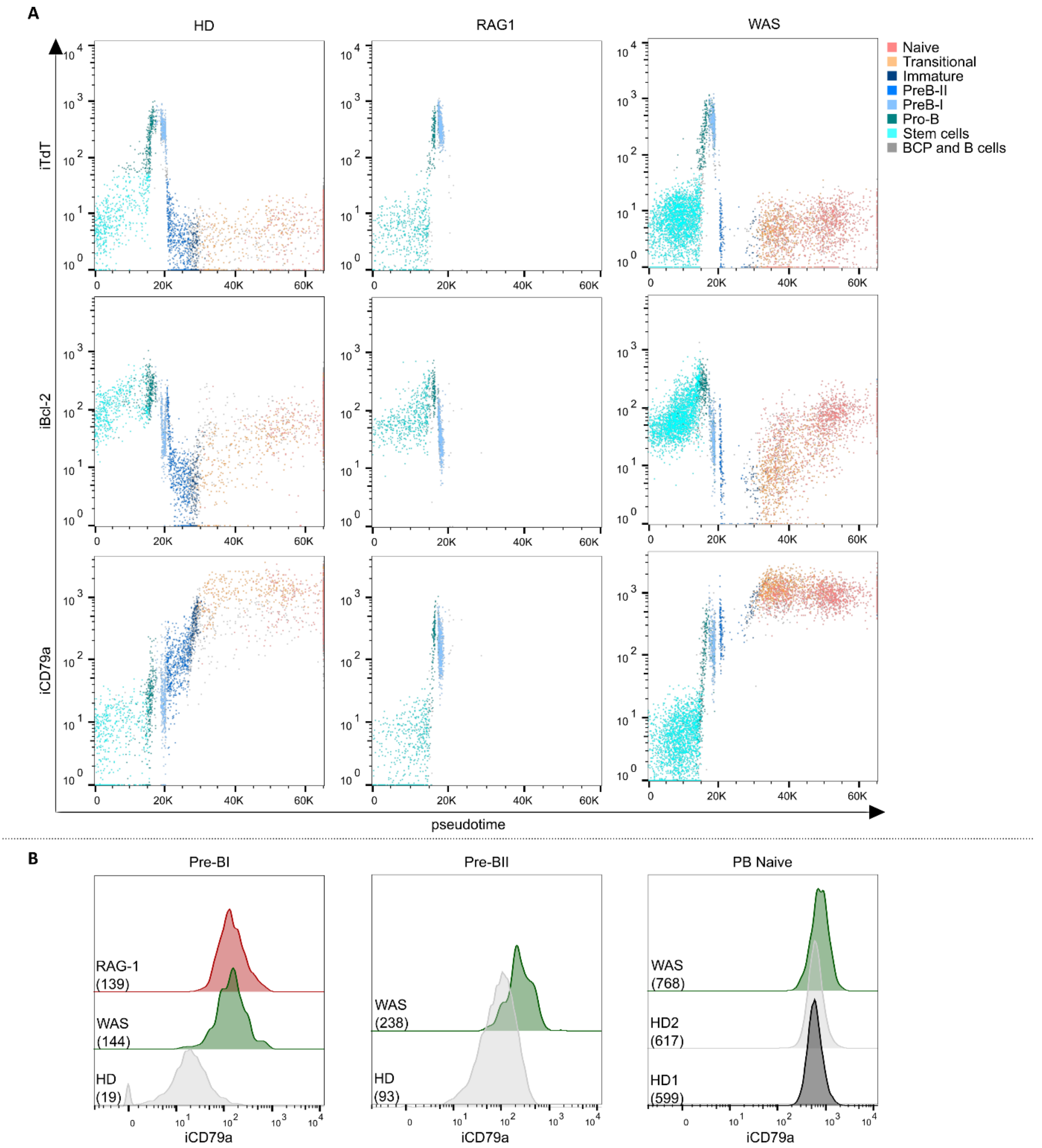

Supplementary Figure 17. (A) Expression of iTdT (top), iBcl-2 (middle) and iCD79a (bottom) along aligned pseudotime of healthy donor (HD), RAG-1 and WAS bone marrow samples with manually defined subpopulations overlaid in color. (B) Histograms showing iCD79a expression in Pre-BI (left), Pre-BII (middle) and peripheral blood Naïve B cells (right) for RAG-1 (red), WAS (green) patients and healthy donor (grey) bone marrow (HD) or peripheral blood samples (HD1, HD2). Median metal intensities are given in brackets.
