# Supplementary material for: Tviblindi algorithm identifies branching developmental trajectories of human B‐cell development and describes abnormalities in RAG‐1 and WAS patients
Source: Eur J Immunol. 2024 Sep 5;54(12):2451004. doi: 10.1002/eji.202451004 (PMC11628918; doi:10.1002/eji.202451004)
Supplement: Supplementary file 2 — SUPPORTING INFORMATION [file EJI-54-2451004-s001.docx]

| marker | clone | tag | µl per 100 µl cell suspension | manufacturer |
| --- | --- | --- | --- | --- |
| **BARCODES** |  |  |  |  |
| CD45 | HI30 | Y89 | 1 | Fluidigm |
| CD45 | MEM-28 | 110Cd | 2 | Exbio |
| CD45 | MEM-28 | 113In | 1 | Exbio |
| HLA-I | W6/32 | 116Cd | 2 | Bxcell |
| HLA-I | W6/32 | 175Lu | 1 | Bxcell |
| **SURFACE MARKERS** |  |  |  |  |
| CD38 | HIT2 | 141Pr | 2 | Exbio |
| sIgM | MHM-88 | 144Nd | 1 | Biolegend |
| CD24 | ML5 | 145Nd | 2 | Biolegend |
| IgD | IA6-2 | 146Nd | 1 | Fluidigm |
| CD20 | 2H7 | 147Sm | 1 | Fluidigm |
| CD34 | 581 | 148Nd | 2 | Fluidigm |
| CD127 | A019D5 | 149Sm | 2 | Fluidigm |
| Ig light chain lambda | MHL-38 | 151Eu | 1 | Fluidigm |
| CD135 | BV10A4 | 156Gd | 4 | Exbio |
| CD10 | HI10a | 158Gd | 1 | Fluidigm |
| CD22 | HIB22 | 159Tb | 2 | Fluidigm |
| Ig light chain kappa | MHK-49 | 160Gd | 1 | Fluidigm |
| CD9 | MEM-61 | 161Dy | 2 | Exbio |
| CD25 | MEM-181 | 162Dy | 4 | Exbio |
| CD44 | MEM-85 | 163Dy | 2 | Exbio |
| CD27 | L128 | 167Er | 1 | Fluidigm |
| CD19 | HIB19 | 169Tm | 2 | Fluidigm |
| CXCR5 | REA103 | 171Yb | 2 | Miltenyi |
| CXCR4 | REA649 | 173Yb | 2 | Miltenyi |
| HLA-DR | L243 | 174Yb | 1 | Fluidigm |
| CD73 | AD2 | 176Yb | 2 | Exbio |
| **INTRACELLULAR MARKERS** |  |  |  |  |
| Caspase 3 (Cleaved) | D3E9 | 142Nd | 2 | Fluidigm |
| cPARP | F21-852 | 143Nd | 1 | Fluidigm |
| PAX-5 | 1H9 | 150Nd | 2 | Biolegend |
| Caspase 7 (Cleaved) | D6H1 | 152Sm | 1 | Fluidigm |
| BCL-2 | Bcl-2/100 | 153Eu | 2 | Exbio |
| Tdt | E17-1519 | 164Dy | 1 | Fluidigm |
| Biotin | 1D4-C5 | 165Ho | 1 | Fluidigm |
| Ki-67 | B56 | 168Er | 1 | Fluidigm |
| CD79a | HM57 | 170Er | 2 | Exbio |
| (iIgM | MHM-88 | 172Yb | 1 | Fluidigm) |
| **LINEAGE NEGATIVE MARKERS** | |  |  |  |
| CD3 | UCHT1 | biotin | 2 | Exbio |
| CD16 | 3G8 | biotin | 2 | Biolegend |
| CD33 | HIM3-4 | biotin | 2 | Exbio |
| CD66b | G10F5 | biotin | 2 | Biolegend |
| **DNA INTERCALATOR** |  |  |  |  |
|  |  | 191Ir/193Ir | 1 | Fluidigm |

**Supplementary Table 1**. Mass cytometry panel of markers used for staining of the samples. The marker in brackets (iIgM) was excluded from the manual analysis due to poor performance.
